# Optimal resistance training strategies for knee osteoarthritis symptom relief: a systematic review and network meta-analysis

**DOI:** 10.1186/s12891-025-09341-0

**Published:** 2025-12-12

**Authors:** Yong Yang, Junyu Wang, Tao Wang, Yuan Yuan, Bopeng Qiu, Wenke Hu, Fuhong Wang, Zhihong Tang, Shu-Cheng Lin

**Affiliations:** 1 Laboratory of Kinesiology and Rehabilitation, School of Physical Education and Sport, Chaohu University, Hefei, China; 2 School of Exercise and Health, Shanghai University of Sport, Shanghai, China; 3 College of Physical Education and Health, Southwest University of Science and Technology, Mianyang, China; 4 Department of Sport Industry Studies, Yonsei University, Seoul, Republic of Korea; 5 School of Strength and Conditioning, Beijing Sport University, Beijing, China; 6 Datuan Community Health Service Center, Shanghai, China; 7 School of Gerontology and Long-Term Care, College of Nursing, Taipei Medical University, Taipei, Taiwan

**Keywords:** Knee osteoarthritis, Resistance training, Middle-aged and older adults; Dose response

## Abstract

**Aims:**

To determine the most effective resistance training (RT) type and the optimal intensity, duration, and number of repetitions for patients with knee osteoarthritis (KOA).

**Methods:**

PubMed, MEDLINE, Embase, CENTRAL, and Web of Science were searched from inception to April 2025 for randomized controlled trials (RCTs) of exercise for KOA. Bayesian network meta-analyses were conducted. The outcomes of pain, stiffness, and function were expressed as standardized mean differences (SMDs) with 95% credible intervals (CrI).

**Results:**

The analysis included 46 RCTs with 3463 participants. High-speed RT was identified as the most effective type, significantly improving pain (SMD: -1.35, 95% CrI: -1.89 to -0.81), stiffness (SMD: -1.26, 95% CrI: -1.81 to -0.70), and function (SMD: 1.70, 95% CrI: 0.96 to 2.45). Dose-response analysis indicated that moderate-intensity RT (43–47% 1RM) for 35–37 weeks with 610–640 weekly repetitions was optimal for reducing pain (SMD: -0.76, 95% CrI: -1.03 to -0.44) and improving function (SMD: 1.29, 95% CrI: 0.92 to 1.68). For stiffness, a higher number of repetitions and shorter duration (12 weeks, 1200 weekly repetitions) were more effective (SMD: -1.11, 95% CrI: -1.58 to -0.64).

**Conclusions:**

This study identifies high-speed RT as the most effective type for managing KOA and provides optimal RT dosages for different symptoms. These findings emphasize the need for tailored RT programs and provide crucial insights for clinical guidelines and individualized patient care.

**PROSPERO number:**

CRD42024535806

**Supplementary Information:**

The online version contains supplementary material available at 10.1186/s12891-025-09341-0.

## Introduction

Knee osteoarthritis (KOA) is a degenerative joint disease characterized by the breakdown of cartilage, leading to pain, stiffness, and decreased function [1]. It predominantly affects older adults, causing significant disability and impacting their quality of life [2]. Globally, KOA is highly prevalent, with over 260 million people affected as of 2017 [3], and its incidence is expected to rise with the aging population. This condition imposes a substantial burden on healthcare systems and personal finances [4], as it often leads to chronic pain management, joint replacement surgeries, and loss of productivity.

Due to its numerous benefits, resistance training (RT) has been widely recommended for managing KOA. Clinical guidelines consistently advise regular strength training as a core treatment for all individuals with KOA, supported by robust evidence from numerous clinical trials [5]. RT primarily includes various forms such as concentric-eccentric isometric training, concentric-eccentric isokinetic training, and concentric-focused training [6, 7]. These forms of RT are known to enhance muscle strength, which is crucial for supporting and stabilizing the knee joint [8], thereby reducing joint load and pressure. This reduction in load helps alleviate pain and discomfort [9]. Additionally, increased muscle strength improves the shock-absorbing capacity of the lower limb muscles during activities such as walking [9], providing extra protection to the knee joint. RT also improves blood circulation, promoting the repair and regeneration of soft tissues and reducing inflammation.

However, there are two significant knowledge gaps in the literature that may hinder the effective use of RT. Firstly, the relationship between RT and KOA symptom improvement may depend on the specific type of RT employed. Various studies have examined different RT modalities, yet the optimal type for maximizing benefits remains unclear. Traditional RT typically includes concentric-eccentric isometric training, but research has shown that concentric-eccentric isokinetic training is more effective in enhancing muscle strength and alleviating symptoms [10, 11]. Additionally, a study indicates that RT focusing on concentric contractions can achieve similar outcomes in pain relief and functional improvement as concentric-eccentric isokinetic training [12]. Evidence indicates that in trained men, eccentric-focused training increases isometric torque and muscle activation more than concentric-focused training [13]; however, subsequent studies have not observed this difference in patients with KOA [14]. Nonetheless, these studies have demonstrated that RT focusing on either eccentric or concentric contractions significantly alleviates pain and enhances physical function in patients with KOA [14–16]. Recent studies have introduced high-speed RT, requiring patients with KOA to perform concentric-eccentric isotonic muscle contractions as quickly as possible. This high-speed approach has proven to be a safe, feasible, and effective method to relieve pain, reduce stiffness, and improve physical function and strength [17–19]. Given the varied forms and outcomes of RT, identifying the most suitable type and dose for patients with KOA remains complex, necessitating further investigation.

Secondly, establishing the dose-response relationship between RT and KOA symptoms is crucial. The World Health Organization’s 2020 guidelines on physical activity and sedentary behavior [20] and the 2018 US Physical Activity Guidelines Advisory Committee [21] highlighted the importance of exploring this relationship. Determining the minimal effective dose, optimal dose, and maximum safe threshold of RT is essential for developing effective training programs for patients with KOA.

Given the current evidence, selecting the optimal RT type for clinicians and patients with KOA can be a complex task. Therefore, this systematic review and network meta-analysis aim to compare the effects of different RT types on pain, stiffness, and function in patients with KOA, providing clearer guidance for clinical decision-making.

## Methods

This pre-registered systematic review and network meta-analysis, with a PROSPERO reference number CRD42024535806, adhered to the reporting requirements outlined in the PRISMA checklist [22].

### Literature search

A comprehensive search was conducted across multiple databases—PubMed, MEDLINE, Embase, Cochrane Central Register of Controlled Trials (CENTRAL), and Web of Science—from their inception until April 2, 2025, without imposing any language restrictions. The detailed search strategies, including search terms, dates, and methodologies, are documented in Supplementary File 1. We also examined the reference lists of pertinent articles and reviews to uncover additional studies. Screening of titles, abstracts, and full texts was independently performed in duplicate by two researchers (YY and JW). Any discrepancies were resolved through discussion or, if necessary, by involving a third author (FW) for final adjudication.

### Eligibility criteria

In accordance with the PICOS approach [23], the inclusion criteria were as follows: (a) participants: Individuals diagnosed with KOA, classified as grade I or higher according to the Kellgren & Lawrence radiological classification [24]; (b) intervention: Based on different contraction forms and contraction speeds, resistance training is categorized into seven distinct types (see Supplementary File 2); (c) comparator: non-physically active (e.g., health education, no intervention, and self-management) control groups; (d) outcomes: studies reporting pain, stiffness, or function, these measures outcomes were mostly based on self-reported pain, function, and stiffness aspects of the Western Ontario and McMaster Universities Osteoarthritis Index (WOMAC) [25]. When more than one scale was presented for pain, stiffness or function, the more comprehensively reported scale was selected in the ranking order proposed by M Fransen, S McConnell, AR Harmer, M Van der Esch, M Simic and KL Bennell [26] and J-P Regnaux, M-M Lefevre-Colau, L Trinquart, C Nguyen, I Boutron, L Brosseau and P Ravaud [27]; (e) study design: included published RCTs [28]. We excluded studies on the acute effects of a single RT session on KOA, and studies that did not clearly describe the muscle contraction forms of RT and specific training variables (e.g., period, frequency, volume, intensity). Studies examining the effects of concurrent training (i.e., combining RT and endurance training) and nutritional supplements in combination with RT were excluded. Studies for postoperative pain and abstract only were also excluded.

### Data extraction

Following the retrieval of all relevant articles from the specified databases, they were cataloged in an EndNote X9 reference manager. Two authors (YY and JW) independently conducted data extraction from studies meeting the inclusion criteria, resolving any discrepancies through consensus among all authors. Pertinent publication details (such as author, title, year, journal), patient numbers, demographics (e.g., age and sex), considered interventions, and outcome measures were extracted. During data extraction, if the original study reported standard errors in the experimental and control groups, standard deviation was computed using the formula:

$$\:\mathrm{S}\mathrm{t}\mathrm{a}\mathrm{n}\mathrm{d}\mathrm{a}\mathrm{r}\mathrm{d}\:\mathrm{d}\mathrm{e}\mathrm{v}\mathrm{i}\mathrm{a}\mathrm{t}\mathrm{i}\mathrm{o}\mathrm{n}\left(\mathrm{S}\mathrm{D}\right)=\mathrm{S}\mathrm{t}\mathrm{a}\mathrm{n}\mathrm{d}\mathrm{a}\mathrm{r}\mathrm{d}\:\mathrm{e}\mathrm{r}\mathrm{r}\mathrm{o}\mathrm{r}\left(\mathrm{S}\mathrm{E}\right)\times\sqrt{\mathrm{n}}$$

In instances where both were unavailable, SD estimation was performed based on parameters like confidence intervals, t-values, quartiles, ranges, or p-values, as outlined in Sect. 7.7.3 of the Cochrane Handbook for Systematic Reviews. If essential data for the study couldn’t be derived through these methods, attempts were made to contact the study authors at least four times within a six-week period to request the required information.

### Risk of bias and quality of evidence

Two authors (YY and JW) independently evaluated the risk of bias in the studies using the revised Cochrane risk of bias tool (RoB 2 tool) at the study level [29]. Any disagreements in data extraction or assessment were resolved by consulting a third author (FW). The confidence of evidence was also evaluated using the CINeMA (Confidence in Network Meta-Analysis) web application, through which the confidence in the results was graded as high, moderate, low, or very low [30].

### Data synthesis

A network plot was generated to visually represent the network of comparisons across trials, ensuring the viability of the network meta-analyses. Bayesian network meta-analyses were conducted using the ‘gemtc’ and ‘rjags’ packages within the R statistical environment (V.4.2.2, www.r-project.org). This approach involves calculating the posterior distribution of parameters based on the available data to update prior information, as Bayesian methods are more prevalent in such analyses compared to frequentist approaches. Markov chains were used to generate samples. Model convergence was assessed using the Brooks-Gelman-Rubin plots method. The effect sizes were calculated as standardized mean differences (SMD) of the change score because the studies use different rating scales or units of the outcome. The amount of baseline and post-training change between the experimental group and control group were calculated by the following formula:

$$\:\mathrm{M}\mathrm{e}\mathrm{a}{\mathrm{n}}_{\mathrm{c}\mathrm{h}\mathrm{a}\mathrm{n}\mathrm{g}\mathrm{e}}=\mathrm{M}\mathrm{e}\mathrm{a}{\mathrm{n}}_{\mathrm{p}\mathrm{o}\mathrm{s}\mathrm{t}\mathrm{t}\mathrm{r}\mathrm{a}\mathrm{i}\mathrm{n}\mathrm{i}\mathrm{n}\mathrm{g}}-\mathrm{M}\mathrm{e}\mathrm{a}{\mathrm{n}}_{\mathrm{b}\mathrm{a}\mathrm{s}\mathrm{e}\mathrm{l}\mathrm{i}\mathrm{n}\mathrm{e}}$$

$$\:\mathrm{S}{\mathrm{D}}_{\mathrm{c}\mathrm{h}\mathrm{a}\mathrm{n}\mathrm{g}\mathrm{e}}=\sqrt{\mathrm{S}{\mathrm{D}}_{\mathrm{b}\mathrm{a}\mathrm{s}\mathrm{e}\mathrm{l}\mathrm{i}\mathrm{n}\mathrm{e}}^{2}+\mathrm{S}{\mathrm{D}}_{\mathrm{p}\mathrm{o}\mathrm{s}\mathrm{t}\mathrm{t}\mathrm{r}\mathrm{a}\mathrm{i}\mathrm{n}\mathrm{i}\mathrm{n}\mathrm{g}}^{2}-\left(2\times\mathrm{r}\times\mathrm{S}{\mathrm{D}}_{\mathrm{b}\mathrm{a}\mathrm{s}\mathrm{e}\mathrm{l}\mathrm{i}\mathrm{n}\mathrm{e}}\times\mathrm{S}{\mathrm{D}}_{\mathrm{p}\mathrm{o}\mathrm{s}\mathrm{t}\mathrm{t}\mathrm{r}\mathrm{a}\mathrm{i}\mathrm{n}\mathrm{i}\mathrm{n}\mathrm{g}}\right)}$$

Where *r* is a constant (*r* = 0.5), which provides a conservative estimate [31]. To evaluate the reliability of our estimates, we utilized 95% credible intervals (CrI). Clinically, an SMD (standardized mean difference) of 0.20 is considered a small effect, 0.50 a moderate effect, and 0.80 a large effect, as defined by Cohen [32]. For data synthesis, a random-effects model was employed to combine the data, while the surface under the cumulative ranking (SUCRA) probabilities was utilized to rank the various treatments. We assessed the transitivity assumption by comparing the distribution of potential effect modifiers (publication year, mean age, sample size, percentage of male participants, and disease grade) among studies grouped by comparison (see Supplementary File 3). Statistical heterogeneity between studies was examined using the tau-square (τ^2^) test and I^2^ statistics. Statistical consistency was evaluated using the design-by-treatment test [33] and by differentiating indirect from direct evidence (SIDE test) [34] via the R ‘netmeta’ package. We further conducted a comparison using adjusted funnel plots to assess potential publication bias under specific circumstances [35]. Egger’s test was used to detect potential publication bias, considering *p* < 0.05 as statistically significant.

The ‘MBNMAdose’ package based on R statistical environment (V.4.2.2, R Core Team, www.r-project.org) was used to perform random-effects Bayesian Model-Based Network Meta-Analysis (MBNMA) [36] to summarize the dose-response association between RT dose (intensity, period and the number of repetitions/week) and outcomes. First, the connectivity is a key assumption in network meta-dose analysis, and evidence of un-connectedness may lead to low statistical power and misleading results [37]. By drawing treatment-level plots, we verified that the connectivity in this study (Supplementary File 11). We analyzed the data with the consistency model and the unrelated mean effect model, and compared the differences in the deviation, the number of estimated parameters in the network, and the Deviance Informative Criterion (DIC) indicators of the two models. If these are similar, it means that our research has good consistency (Supplementary File 11, Table 11.1) [38]. We assessed transitivity via MBNMA node-splitting approach. This method splits and compares contributions for a particular treatment contrast into direct and indirect evidence [39]. Similar effects denote good transitivity (Supplementary File 11, Table 11.2.1–9.1). In order to better find the relationship between RT dose (intensity, period and the number of repetitions/week) and outcomes, a series of nonlinear and linear models were used. Next, we derived and compared different fit indices and corresponding deviance plots across all estimated models [40]. Restricted cubic splines were used for all outcomes, except that a linear model was applied for the relationship between stiffness and the number of repetitions per week(Supplementary File 12, Table 12.1-9.1).

## Results

### Study selection

**Fig. 1:**
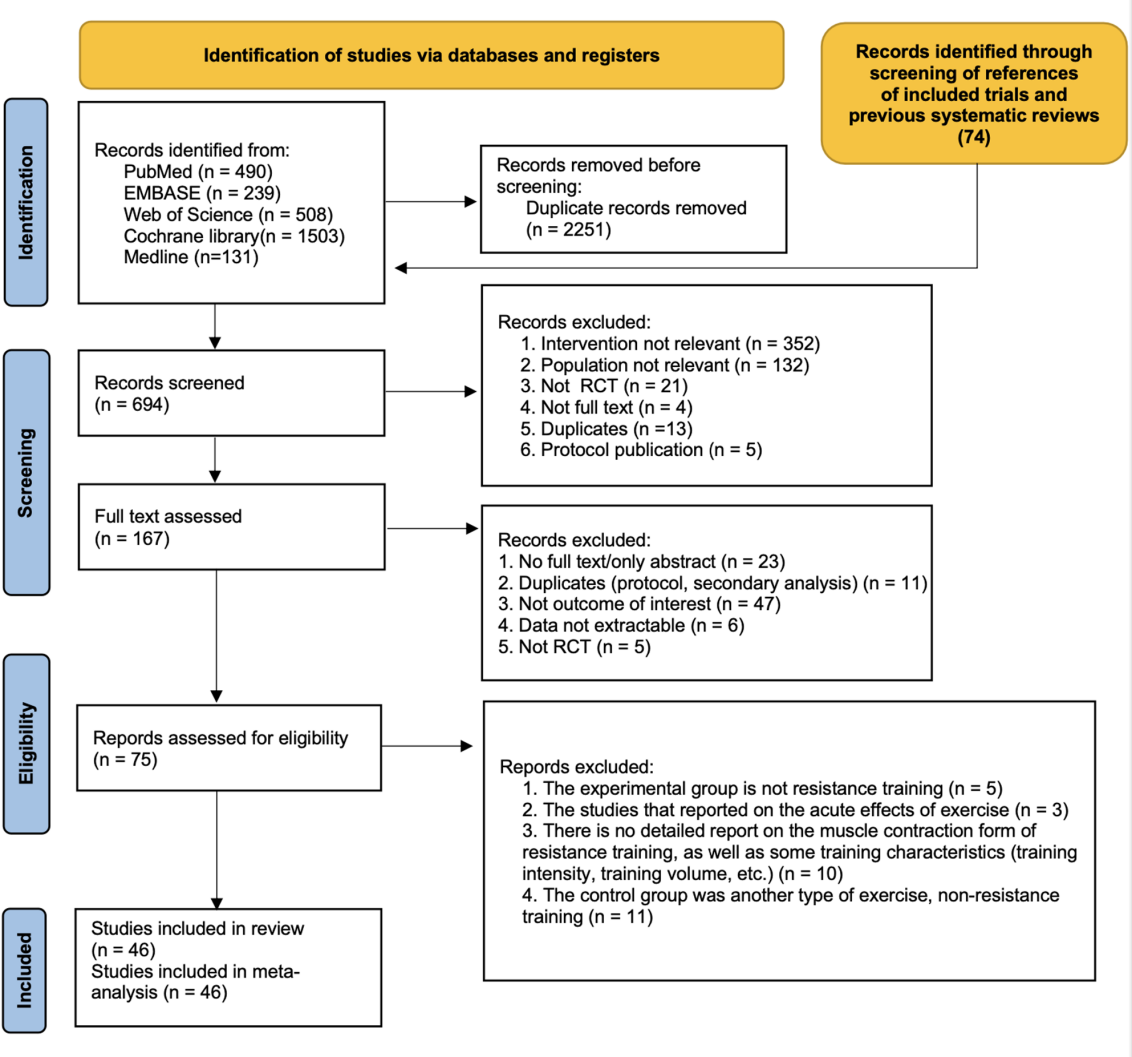
PRISMA flow diagram of the search process for included studies

Overall, 2871 records were identified through the initial electronic searches. After removing duplicates, 703 records were screened for titles and abstracts and 176 full-text articles were screened for eligibility. In total, 46 studies involving 3463 participants were included in the review (Fig. 1).

### Characteristics of included studies

The characteristics of included studies were shown in (Table 1). A total of 196 participants across 5 studies underwent combined concentric-eccentric isotonic training in water (ACCEIT), 24 studies with 1059 participants underwent combined concentric-eccentric isotonic training (CCEIT), 5 studies with 129 participants underwent concentric isotonic training (CIT), 3 studies with 77 participants underwent eccentric isotonic training (EIT), 3 studies with 49 participants underwent combined concentric-eccentric isotonic training, as fast as possible (High speed), 9 studies with 217 participants underwent combined concentric-eccentric isokinetic training (Isokinetic), 9 studies with 369 participants underwent isometric muscle contraction (Isometric), and total 1367 participants underwent usual care, health education, or no intervention, these conditions were collectively classified as the control group (CON). The sample size of the included studies ranged from 14 to 251, with a median of 57. The mean age ranged from 53.4 to 74.5, with a median of 63.7. The disease grade ranged from 1.37 to3.5, with a median of 2.14. The year of publication ranged from 1996 to 2022, with a median of 2013. The RT adherence was 72.2–100%. The RT intervention lasted for 4 to 72 weeks, with a training frequency of 2 to 14 times/week, 1–10 sets per exercise, 5–20 repetitions per set, and 30–180 s of rest between sets. The training intensity ranged from 10% to 85% of 1RM (Supplementary File 4).

**Table 1:** Characteristics of subjects included in the review (*n* = 46)

Study	RT	CON	*N*(M (RT vs. CON)	Age (RT vs. CON)	K/L grade (RT vs. CON)	Drop out (RT vs. CON)	RT Adherence	Pain	Stiffness	Function
Oliveira et al. (2012)	Concentric Isotonic Training	Usual care	50(3) vs. 50(3)	61.50 ± 6.94 vs. 58.78 ± 9.60	2.10 ± 0.36 vs. 2.10 ± 0.36	7 vs. 12	98%	WOMAC pain, score	WOMAC stiffness, score	WOMAC function, score
de Almeida et al. (2020)	RT1: Combined Concentric-Eccentric Isotonic Training, as fast as possible RT2: isometric muscle contraction	Health education	20(5) vs. 21(5) vs. 20(4)	55.6 ± 5.3 vs. 55.2 ± 7.4 vs. 53.8 ± 7.7	2.2 ± 0.41 vs. 2.29 ± 0.46 vs. 2.25 ± 0.44	2 vs. 1 vs. 2	100%	VAS, score	NA	NA
de Almeida et al. (2021)	RT1: Combined Concentric-Eccentric Isotonic Training, as fast as possible RT2: isometric muscle contraction	Health education	20(5) vs. 21(5) vs. 20(4)	55.6 ± 5.3 vs. 55.2 ± 7.4 vs. 53.8 ± 7.7	2.2 ± 0.41 vs. 2.29 ± 0.46 vs. 2.25 ± 0.44	2 vs. 1 vs. 2	100%	WOMAC pain, score	WOMAC stiffness, score	WOMAC function, score
Waller et al. (2017)	Combined Concentric-Eccentric Isotonic Training in Water	Usual care	43(0) vs. 44(0)	63.8 ± 2.4 vs. 63.9 ± 2.4	1.47 ± 0.50 vs.1.45 ± 0.50	1 vs. 2	88%	KOOS pain, score	NA	KOOS function, score
Lim et al. (2008)	Combined Concentric-Eccentric Isotonic Training	No intervention	53(23) vs. 54(25)	67.2 ± 6.7 vs. 66.6 ± 8.9	2–4	4 vs. 6	89%	WOMAC pain, score	NA	WOMAC function, score
Gür et al. (2002)	RT1: Combined Concentric-Eccentric Isokinetic Training RT2: Concentric Isotonic Training	No intervention	8(NA) vs. 9(NA) vs. 6(NA)	55 ± 12 vs. 56 ± 12 vs. 57 ± 9	2.45 ± 0.97 vs. 2.35 ± 0.74 vs. 2.45 ± 0.97	No dropouts	100%	NRS pain, score	NA	NRS function, score
Farr et al. (2010)	Combined Concentric-Eccentric Isotonic Training	Self-management	62(13) vs. 57(16)	54.2 ± 7.3 vs. 55.8 ± 6.1	1.44 ± 0.50 vs. 1.40 ± 0.49	12 vs. 6	76%	WOMAC pain, score	NA	NA
Vincent et al. (2019)	RT1: Eccentric Isotonic Training RT2: Concentric Isotonic Training	Wait list	30(10) vs. 28(9) vs. 32(11)	66.8 ± 5.4 vs. 69.5 ± 6.5 vs. 68.6 ± 7.2	2.5	10 vs. 8 vs. 12	96.4% vs. 94.8%	WOMAC pain, score	WOMAC stiffness, score	WOMAC function, score
Pazit et al. (2018)	Combined Concentric-Eccentric Isotonic Training, as fast as possible	No intervention	9(5) vs. 9(4)	67.78 ± 6.28 vs. 70.44 ± 7.83	NA	1 vs. 1	99.3%	WOMAC pain, score	WOMAC stiffness, score	WOMAC function, score
Rogers et al. (2012)	Combined Concentric-Eccentric Isotonic Training	Placebo: Apply cream to knees	8(2) vs. 8(3)	70.8 ± 6.5 vs. 71.2 ± 10.9	NA	No dropouts	96.4%	WOMAC pain, score	WOMAC stiffness, score	WOMAC function, score
Jan et al. (2008)	RT1: Combined Concentric-Eccentric Isotonic Training, high intensity RT2: Combined Concentric-Eccentric Isotonic Training, low intensity	No intervention	34(7) vs. 34(7) vs. 30(5)	63.3 ± 6.6 vs. 61.8 ± 7.1 vs. 62.8 ± 6.3	2.13 ± 0.60 vs. 2.10 ± 0.60 vs. 2.10 ± 0.57	3 vs. 0 vs. 4	88.2% vs. 100%	WOMAC pain, score	NA	WOMAC function, score
Wortley et al. (2013)	Combined Concentric-Eccentric Isotonic Training	Usual care	13(4) vs. 6(2)	69.5 ± 6 vs. 70.5 ± 5	2 ± 0.5 vs. 2 ± 0.25	2 vs. 3	87%	WOMAC pain, score	WOMAC stiffness, score	WOMAC function, score
Foroughi et al. (2011)	Combined Concentric-Eccentric Isotonic Training	sham-exercise program	26(0) vs. 28(0)	66.0 ± 8.0 vs. 65.0 ± 7.0	2.15 ± 0.99 vs. 2.13 ± 1.10	6 vs. 3	97%	WOMAC pain, score	WOMAC stiffness, score	WOMAC function, score
Foroughi et al. (2011)	Combined Concentric-Eccentric Isotonic Training	sham-exercise program	18(0) vs. 19(0)	64.0 ± 7.0 vs. 64.0 ± 8.0	2.67 ± 1.28 vs. 2.26 ± 1.24	8 vs. 9	94%	WOMAC pain, score	WOMAC stiffness, score	WOMAC function, score
DeVita et al. (2018)	Combined Concentric-Eccentric Isotonic Training	no attention control group	15(5) vs. 15(7)	58.1 ± 6.5 vs. 56.2 ± 8.9	2.60 ± 0.91 vs. 2.73 ± 1.03	1 vs. 0	93.8%	WOMAC pain, score	NA	WOMAC function, score
Jorge et al. (2015)	Combined Concentric-Eccentric Isotonic Training	Wait list	29(0) vs. 31(0)	61.7 ± 6.4 vs. 59.9 ± 7.5	1.56 ± 0.51 vs. 1.37 ± 0.50	2 vs. 4	87.5%	WOMAC pain, score	WOMAC stiffness, score	WOMAC function, score
Bruce-Brand et al. (2012)	Combined Concentric-Eccentric Isotonic Training	Standard care	10(6) vs. 6(3)	63.4 ± 5.9 vs. 65.2 ± 3.1	3.5	3 vs. 4	83%	WOMAC pain, score	WOMAC stiffness, score	WOMAC function, score
Topp et al. (2002)	RT1: Combined Concentric-Eccentric Isotonic Training RT2: isometric muscle contraction	No intervention	32(11) vs. 35(10) vs. 35(7)	63.53 ± 1.90 vs. 65.57 ± 1.82 vs. 60.94 ± 1.82	NA	No dropouts	NA	WOMAC pain, score	WOMAC stiffness, score	WOMAC function, score
Isaramalai et al. (2018)	RT1: isometric muscle contraction, based on self-weight RT2: isometric muscle contraction, based on sandbags	standard treatment	25(3) vs. 25(2) vs. 25(5)	68.0 ± 5.80 vs. 68.0 ± 5.80 vs. 63.7 ± 4.30	1.92 ± 0.91 vs. 1.92 ± 0.89 vs. 2.12 ± 0.83	No dropouts	NA	WOMAC pain, score	WOMAC stiffness, score	WOMAC function, score
Oh et al. (2021)	Combined Concentric-Eccentric Isotonic Training	Health education	21(NR) vs. 11(NR)	71.05 ± 6.45 vs. 70.55 ± 4.80	≤ 2	19 vs. 9	≥ 80%	WOMAC pain, score	WOMAC stiffness, score	WOMAC function, score
Assar et al. (2020)	Combined Concentric-Eccentric Isotonic Training	No exercise intervention	12(NR) vs. 12(NR)	55.9 ± 8.6 vs. 63.8 ± 7.5	2.7 ± 0.8 vs. 2.7 ± 0.6	No dropouts	NA	WOMAC pain, score	WOMAC stiffness, score	NA
Messier et al. (2021)	RT1: Combined Concentric-Eccentric Isotonic Training, high intensity RT2: Combined Concentric-Eccentric Isotonic Training, low intensity	Health education	127(75) vs. 126(75) vs. 124(76)	67.0 ± 9.0 vs. 64.0 ± 8.0 vs. 64.0 ± 7.0	1.55 ± 0.65 vs. 1.6 ± 0.98 vs. 1.75 ± 0.96	18 vs. 18 vs. 21	78% vs. 77%	WOMAC pain, score	NA	WOMAC function, score
Chang et al. (2012)	Combined Concentric-Eccentric Isotonic Training	Conventional modality treatments	24(0) vs. 17(0)	65.0 ± 8.4 vs. 70.8 ± 8.4	2.63 ± 0.49 vs. 2.82 ± 0.39	1 vs. 3	96%	WOMAC pain, score	WOMAC stiffness, score	WOMAC function, score
Baker et al. (2001)	Combined Concentric-Eccentric Isotonic Training	Health education	23(6) vs. 23(4)	69 ± 6 vs. 68 ± 6	3	4 vs. 3	84%	WOMAC pain, score	NA	WOMAC function, score
Salli et al. (2010)	RT1: Combined Concentric-Eccentric Isokinetic Training RT2: isometric muscle contraction	Usual care	23(4) vs. 24(4) vs. 24(5)	55.73 ± 8.23 vs. 57.1 ± 6.75 vs. 58.3 ± 6.67	NA	2 vs. 1 vs. 1	NA	WOMAC pain, score	NA	WOMAC function, score
Lin et al. (2009)	Combined Concentric-Eccentric Isotonic Training	No intervention	36(12) vs. 36(10)	61.6 ± 7.2 vs. 62.2 ± 6.7	2.56 ± 0.50 s. 2.53 ± 0.50	2 vs. 3	NA	WOMAC pain, score	NA	WOMAC function, score
Jan et al. (2009)	RT1: Combined Concentric-Eccentric Isotonic Training, based on machine RT2: Combined Concentric-Eccentric Isotonic Training, based on self-weight	No exercise intervention	36(12) vs. 35(10) vs. 35(11)	62.0 ± 6.7 vs. 63.2 ± 6.8 vs. 62.2 ± 6.7	2.26 ± 0.60 vs. 2.19 ± 0.64 vs. 2.21 ± 0.61	3 vs. 2 vs. 4	NA	NA	NA	WOMAC function, score
Bennell et al. (2010)	Combined Concentric-Eccentric Isotonic Training	No intervention	45(22) vs. 44(24)	64.5 ± 9.1 vs. 64.6 ± 7.6	3.00 ± 0.83 vs. 3.00 ± 0.84	6 vs. 7	96%	WOMAC pain, score	NA	WOMAC function, score
O’Reilly et al. (1999)	Combined Concentric-Eccentric Isotonic Training	No intervention	108(38) vs. 72(23)	61.94 ± 10.01 vs. 62.15 ± 9.73	NA	6 vs. 5	NA	WOMAC pain, score	NA	WOMAC function, score
Schilke et al. (1996)	Combined Concentric-Eccentric Isokinetic Training	Continued their usual activities	10 (NR) vs. 10 (NR)	64.5 ± 12.10 vs. 68.4 ± 25.81	NA	No dropouts	NA	OASI pain, score	OASI stiffness, score	OASI mobility, score
Rafiq et al. (2021)	Combined Concentric-Eccentric Isotonic Training	Instruction of daily care	25(11) vs. 25(12)	53.40 ± 5.18 vs. 52.84 ± 5.74	2.5	4 vs. 4	72.2%	WOMAC pain, score	WOMAC stiffness, score	WOMAC function, score
Foley et al. (2003)	RT1: Combined Concentric-Eccentric Isotonic Training in water RT2: Combined Concentric-Eccentric Isotonic Training, based on machine	No intervention	35 (20) vs. 35 (15) vs. 35 (15)	73.0 (8.2) vs. 69.8 (9.2) vs. 69.8 (9.0)	NA	7 vs. 9 vs. 3	84% vs. 75%	WOMAC pain, score	WOMAC stiffness, score	WOMAC function, score
Chen et al. (2019)	RT1: Combined Concentric-Eccentric Isokinetic Training RT2: isometric muscle contraction	NA	30(11) vs. 30(18)	65.9 ± 2.9 vs. 65.0 ± 3.1	1.66 ± 0.80 vs. 1.47 ± 0.82	6 vs. 4	83% vs. 88%	WOMAC pain, score	WOMAC stiffness, score	WOMAC function, score
Evcik and Sonel (2002)	Isometric muscle contraction	Continue their normal daily activities	27 (9) vs. 26 (8)	56.3 ± 6.1 vs. 55.8 ± 6.9	1–3	No dropouts	NA	WOMAC pain, score	NA	WOMAC function, score
Vassão et al. (2021)	Combined Concentric-Eccentric Isotonic Training	Not submitted to any kind of therapeutical intervention	13 (0) vs. 10 (0)	62.29 ± 4.39 vs. 66.5 ± 4.06	2.5	1 vs. 4	NA	WOMAC pain, score	WOMAC stiffness, score	WOMAC function, score
Munukka et al. (2020)	Combined Concentric-Eccentric Isotonic Training in water	Usual care	43 (0) vs. 44 (0)	64 (2) vs. 64 (2)	1.5	1 vs. 1	88%	WOMAC pain, score	WOMAC stiffness, score	WOMAC function, score
Silva et al. (2008)	RT1: Combined Concentric-Eccentric Isotonic Training in water RT2: Combined Concentric-Eccentric Isotonic Training, based on self-weight	NA	32(2) vs. 32(3)	59 ± 7.6 vs. 59 ± 6.08	NA	No dropouts	96% vs. 81%	VAS pain, score	NA	NA
Chen et al. (2014)	Combined Concentric-Eccentric Isokinetic Training	No intervention	30(NA) vs. 30(NA)	63.0 ± 7.4	NA	No dropouts	90.0%	VAS pain, score	ROM stiffness	NA
Huang et al. (2003)	RT1: Combined Concentric-Eccentric Isokinetic Training RT2: Combined Concentric-Eccentric Isotonic Training RT3: Isometric Training	No intervention	33(NA) vs. 33(NA) vs. 33(NA) vs. 33(NA)	> 40	2	No dropouts	88% vs. 93% vs. 93%	VAS pain, score	NA	NA
Huang et al. (2005)	Combined Concentric-Eccentric Isokinetic Training	No intervention	35(NA) vs. 35(NA)	65.0 ± 6.4 vs. 65.0 ± 6.4	2	No dropouts	NA	VAS pain, score	ROM stiffness	NA
Huang et al. (2018)	Isometric Training	Usual care	128(27) vs. 122(24)	68.07 ± 9.16 vs. 67.42 ± 7.29	2.77 ± 0.32 vs. 2.78 ± 0.29	No dropouts	NA	VAS pain, score	NA	NA
Munukka et al. (2016)	Combined Concentric-Eccentric Isotonic Training in water	Usual care	43(0) vs. 44(0)	64 ± 2 vs. 64 ± 2	1.47 ± 0.12 vs. 1.45 ± 0.13	1 vs. 2	88%	KOOS pain, score	NA	KOOS function, score
Samut et al. (2015)	Combined Concentric-Eccentric Isokinetic Training	Usual care	15(NA) vs. 13(NA)	62.46 ± 7.71 vs. 60.92 ± 8.85	1.5 ± 0.5 vs. 1.5 ± 0.5	2 vs. 0	NA	WOMAC pain, score	WOMAC stiffness, score	WOMAC function, score
Weng et al. (2009)	Combined Concentric-Eccentric Isokinetic Training	No intervention	33(NA) vs. 33(NA)	64.0 ± 7.5 vs. 64.0 ± 7.5	2	2 vs. 2	NA	VAS pain, score	ROM stiffness	NA
Vincent and Vincent (2020)	RT1: Eccentric Isotonic Training RT2: Concentric Isotonic Training	Continue their normal daily activities	19(6) vs. 17(6) vs. 17(6)	66.8 ± 5.4 vs. 69.5 ± 6.5 vs. 68.6 ± 7.1	2 to 3	11 vs. 11vs. 12	NA	NRS pain, score	NA	NA
Trojani et al. (2022)	RT1: Eccentric Isotonic Training RT2: Concentric Isotonic Training	NA	28(12) vs. 25(4)	74.5 ± 8.3 vs. 72 ± 6.8	2 to 3	2 vs. 5	NA	WOMAC pain, score	WOMAC stiffness, score	WOMAC function, score

*RT* resistance training, *CON* control group, *NA* None-available, *K/L* the Kellgren-Lawrence scale, *VAS* Visual Analogue Scale, WOMAC the Western Ontario and McMaster Universities Osteoarthritis Index, *KOOS* knee injury and osteoarthritis outcome score, *NRS* Numerical Pain Rating scale, *ROM* range of motion

**Fig. 2:**
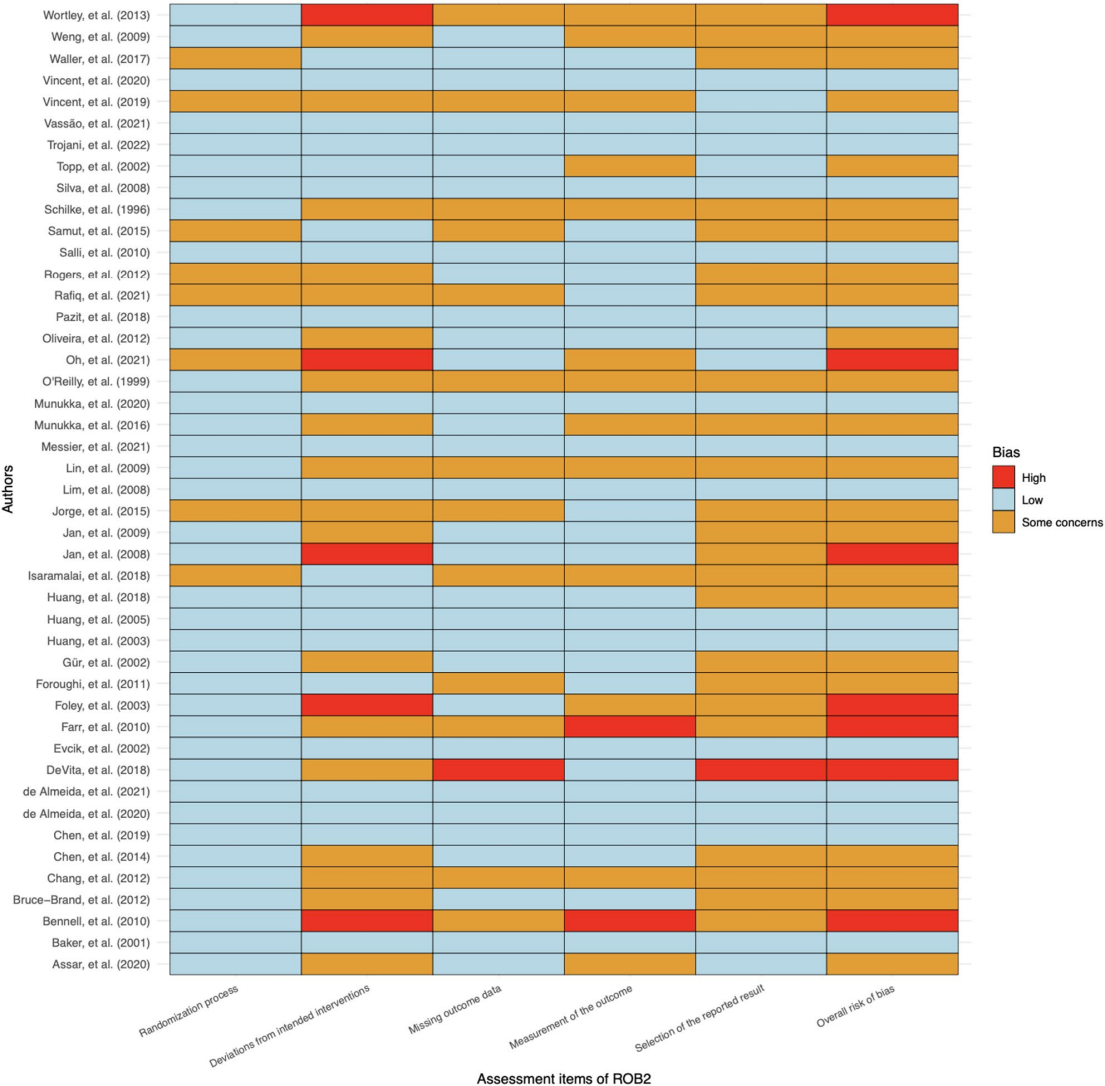
Cochrane Risk of Bias 2 Items Heat Map for included studies

Of the 46 trials, for overall bias, 16 studies were assessed as low risk of bias, 23 as some concerns and 7 as high. In the randomization process, 38 trials were at low risk of bias, 8 trials were at some concerns risk of bias; for deviations from intended interventions, 22 trials were at low risk of bias, 19 trials were at some concerns risk of bias, 5 trials were at high risk of bias; in the missing outcome data, 31 trials were at low risk of bias, 14 trials were at some concerns risk of bias, and 1 trials were at high risk of bias; in the measurement of the outcome, 30 trials were at low risk of bias, 14 trial was at some concerns risk of bias, 2 trials were at high risk of bias; in the selection of the reported result, 21 trials were at low risk of bias, 24 trials were at some concerns risk of bias, 1 trials were at high risk of bias (Fig. 2, Supplementary File 5).

### Network meta-analysis

Heterogeneity results showed low to moderate (I^2^ = 20.5–68.0%). The results of design-by-treatment interaction test showed that global inconsistency was not significant (*P* < 0.05). The SIDE test showed that the percentage of comparisons with evidence of inconsistency was 3.6–7.2% (Supplementary File 6). Additionally, our comparison-adjusted funnel plot had good symmetry for all outcomes, and the results of Egger’s test (*p* < 0.05) showed that no small study effect was found (Supplementary File 9). The confidence of the evidence of 75.0% for pain was very low, 14.3% was low, and 10.7% was moderate. For stiffness, 67.9% was very low, and 32.1% was moderate. For function, 71.4% was very low, 21.4% was low, and 7.2% was moderate (Supplementary File 10).

**Fig. 3:**
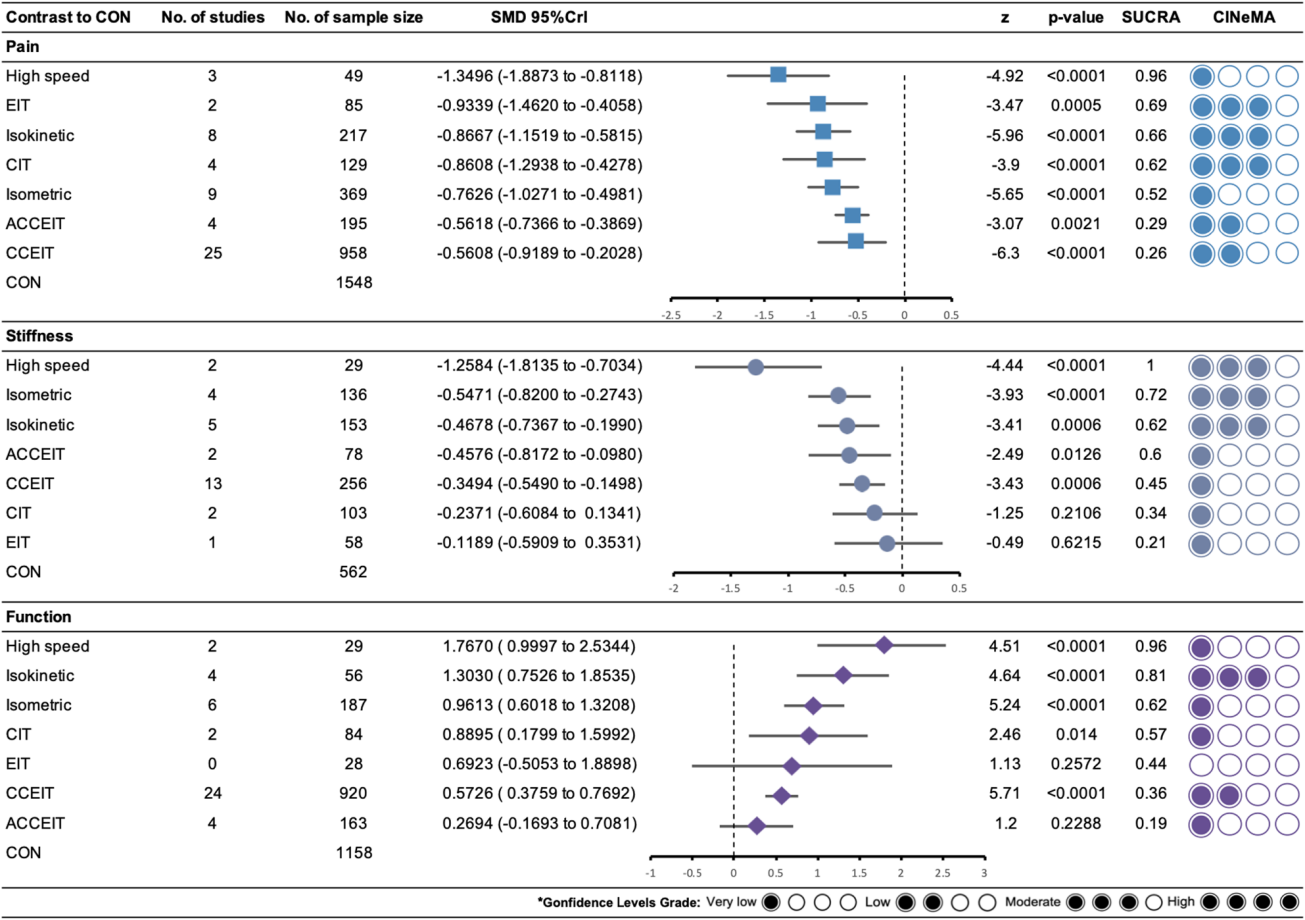
The results of network meta-analysis. ACCEIT, combined concentric-eccentric isotonic training in water; CCEIT, combined concentric-eccentric isotonic training; CIT, concentric isotonic training; EIT, eccentric isotonic training; High speed, combined concentric-eccentric isotonic training, as fast as possible; Isokinetic, combined concentric-eccentric isokinetic training; isometric, isometric muscle contraction

Supplementary file 7 showed the direct comparison and sample size distribution between the RT types for pain. 45 of the studies with 3357 participants assessed pain. Compared with the CON, 7 (100%) of 7 RT types significantly relieved symptoms of pain, and the SMD (95% CrI) ranged between − 1.35 (−1.89; −0.81) for High speed to −0.56 (−0.74; −0.39) for CCEIT (Fig. 3). The results of pairwise comparisons showed that High speed was significantly better than Isometric, ACCEIT, and CCEIT in relieving pain symptoms, and High speed ranks first (SUCRA: 0.96) (Supplementary File 8, Table 8.1). In addition, High speed, EIT, Isokinetic, and CIT all showed large effect size (SMD > 0.8).

Supplementary file 7 showed the direct comparison and sample size distribution between the RT types for stiffness. 26 of the studies with 1350 participants assessed stiffness. Compared with the CON, 5 (71.4%) of 7 RT types significantly relieved symptoms of stiffness, and the SMD (95% CrI) ranged between − 1.26 (−1.81; −0.70) for High speed to −0.35 (−0.55; −0.15) for CCEIT. The results of pairwise comparisons showed that High speed was significantly better than all other RT types in relieving stiffness symptoms, and High speed ranks first (SUCRA: 1.00) (Supplementary File 8, Table 8.2). In addition, only High speed showed large effect size (SMD > 0.8).

Supplementary file 7 showed the direct comparison and sample size distribution between the RT types for function. 35 of the studies with 2411 participants assessed function. Compared with the CON, 5 (71.4%) of 7 RT types significantly improved function, and the SMD (95% CrI) ranged between 1.77 (1.00; 2.53) for High speed to 0.57 (0.38; 0.77) for CCEIT. The results of pairwise comparisons showed that High speed, Isokinetic, and Isometric were significantly better than other RT types in improving function, and High speed ranks first (SUCRA: 0.96) (Supplementary File 8, Table 8.3). In addition, High speed, Isokinetic, CIT, and Isometric all showed large effect size (SMD > 0.8).

### Dose-response relationships

**Fig. 4:**
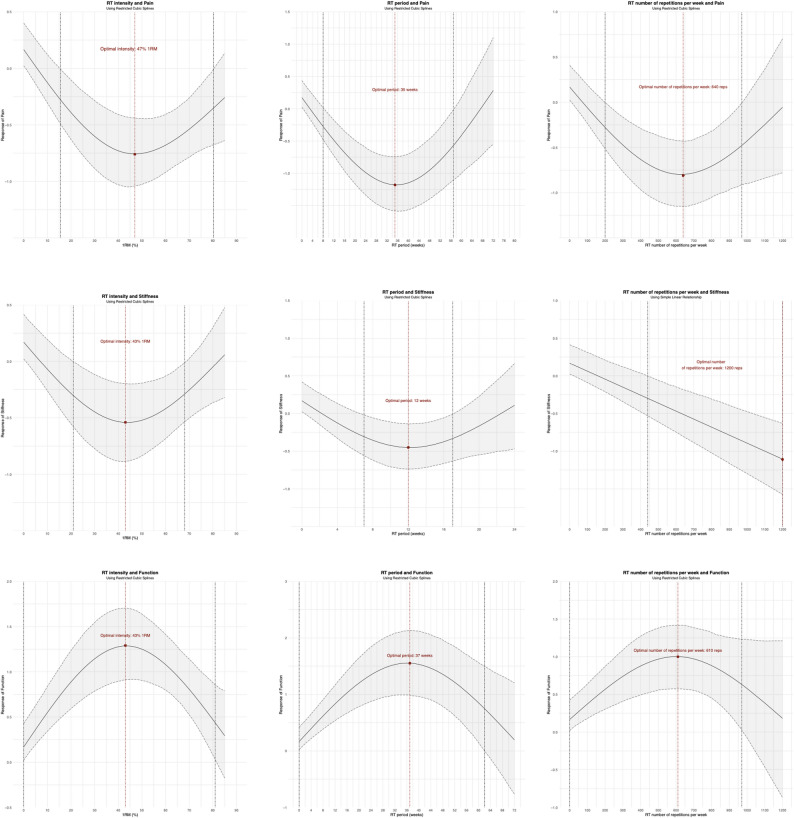
The results of dose-response analysis

Figure 4 demonstrated the dose-response relationships between different RT-specific variables and outcomes of interest, where the shades of green represent the sample size for distributing RT doses, with larger sample sizes being darker and vice versa. We detected U-shaped dose-response relationship between RT intensity (Beta 1: −1.55 (−1.87 to −1.24); Beta 2: 0.142 (−0.33 to 0.58)), period (Beta 1: −1.99 (−2.69 to −1.29); Beta 2: 1.03 (0.20 to 1.86)), and number of repetitions/week (Beta 1: −1.56 (−2.08 to −1.04); Beta 2: 0.40 (−0.42 to 1.21)) and pain symptoms. We observed that 16–80% 1RM was the intensity range, 8–57 weeks was the period range, and 200–970 was the number of repetitions/week range in which RT appears to significantly relieve pain symptoms in the patients with KOA. The predicted maximal significant response was observed at 47% 1RM (SMD: −0.76; 95% CrI (−1.03 to −0.44)), 35 weeks (SMD: −1.18; 95% CrI (−1.59 to −0.75)), and 640 number of repetitions/week (SMD: −0.81; 95% CrI (−1.14 to −0.44)).

We also detected U-shaped dose-response relationship between RT intensity (Beta 1: −1.12 (−1.52 to −0.77); Beta 2: 0.35 (−0.14 to 0.85)), and period (Beta 1: −0.96 (−1.38 to −0.59); Beta 2: 0.35 (−0.25 to 0.96)) and stiffness symptoms. Furthermore, the number of repetitions/week showed a simple linear relationship with symptoms of stiffness (Beta 1: −0.106 (−0.143 to −0.071), the beta value corresponding to each additional 100 repetitions). We observed that 21–68% 1RM was the intensity range, 7–17 weeks was the period range, and more than 440 repetitions per week in which RT appears to significantly relieve stiffness symptoms in the patients with KOA. The predicted maximal significant response was observed at 43% 1RM (SMD: −0.54; 95% CrI (−0.89 to −0.19)), 12 weeks (SMD: −0.45; 95% CrI (−0.74 to −0.14)), and 1200 number of repetitions/week (SMD: −1.11; 95% CrI (−1.58 to −0.64)).

We detected that the intensity (Beta 1: 1.75 (1.31 to 2.21); Beta 2: −0.63 (−1.25 to −0.01)), period (Beta 1: 2.11 (1.08 to 3.10); Beta 2: −0.91 (−1.96 to 0.12)), and number of repetitions per week (Beta 1:1.27 (0.58 to 1.93); Beta 2: −0.55 (−1.77 to 0.63)) of RT showed an inverted U-shaped dose-response relationship with function in patients with KOA. We observed that training intensity ≤ 81% of 1RM, the period ≤ 62 weeks, and the number of repetitions/week ≤ 970 in which RT appears to significantly improve function in the patients with KOA. The predicted maximal significant response was observed at 43% 1RM (SMD: 1.29; 95% CrI (0.92 to 1.68)), 37 weeks (SMD: 1.55; 95% CrI (0.98 to 2.12)), and 610 number of repetitions/week (SMD: 1.00; 95% CrI (0.58 to −1.42)).

## Discussion

This study revealed several key findings regarding the effectiveness of RT in managing KOA. Firstly, among the various RT types, high-speed RT demonstrated the most significant improvements in alleviating pain and stiffness, as well as enhancing physical function. This form of training, which involves performing concentric muscle contractions at maximum speed, proved to be superior in achieving these outcomes compared to other RT modalities. Additionally, the study found different dose-response relationships for improving various KOA symptoms. Distinct symptoms required tailored exercise doses for optimal relief. For instance, the optimal dose for reducing pain was identified as 47% of 1RM performed over 35 weeks, with a weekly frequency of 640 repetitions. In contrast, alleviating stiffness necessitated a higher frequency and shorter duration, with the best results observed at 43% 1RM over 12 weeks, with 1200 weekly repetitions. Functional improvements were maximized at 43% 1RM, performed over 37 weeks, with 610 weekly repetitions. These findings underscore the necessity of personalized RT programs tailored to the unique needs of patients with KOA. By identifying the specific types and doses of RT that yield the greatest benefits for pain, stiffness, and function, this study provides critical insights for developing more effective and targeted interventions.

RT has been widely recognized for its numerous benefits in treating KOA [8, 9, 41]. Multiple studies have demonstrated that RT can significantly reduce pain, enhance physical function, and decrease stiffness in patients with KOA. This study, through a network meta-analysis, found that among the seven RT modalities included, high-speed RT consistently ranked first in improving pain, stiffness, and physical function in patients with KOA. These findings are consistent with previous literature. For example, study showed that high-speed RT significantly reduced pain and improved physical function outcomes in patients with KOA [42]. Similarly, another study reported that high-speed RT was more effective than traditional RT in alleviating stiffness and enhancing knee function [43]. The superior efficacy of high-speed RT can be attributed to several factors. High-speed RT involves performing concentric-eccentric isotonic muscle contractions at maximum speed, which maximizes muscle activation and improves neuromuscular efficiency [44, 45]. This type of training enhances the rapid force production capability of the muscles, which is crucial for activities requiring quick and powerful movements. Additionally, high-speed RT can improve the proprioceptive abilities of the knee joint, leading to better joint stability and control [46, 47]. This enhanced stability reduces the load on the joint, thereby alleviating pain and discomfort. Furthermore, the dynamic nature of high-speed RT may lead to greater improvements in muscle strength and endurance, which are essential for supporting and stabilizing the knee joint, thus reducing stiffness and enhancing overall function [48].

However, the findings of this study reveal that the relationship between RT and symptom improvement in patients with KOA is not simply linear but exhibits a U-shaped curve. This finding indicates that there is an optimal dosage range for RT to be effective in alleviating pain, reducing stiffness, and enhancing physical function. Exercising too little or too much can lead to suboptimal outcomes. The U-shaped relationship may be attributed to the balance between sufficient muscle stimulation and the risk of overtraining. The discovery of this U-shaped relationship holds significant implications for the design of RT programs for patients with KOA. It underscores the necessity of balancing the dosage and intensity of RT to avoid undertraining or overtraining [49]. By doing so, the therapeutic benefits of RT can be maximized, providing a clear guideline for clinicians to develop effective and safe RT protocols tailored to the needs of patients with KOA.

The further dose-response analysis in this study found that the optimal intensity for improving KOA symptoms such as pain, stiffness, and physical function is remarkably similar. Specifically, the optimal intensity for reducing pain was identified at 47% 1RM, while for alleviating stiffness and improving physical function, it was 43% 1RM. These findings are consistent with existing literature, which suggests that moderate-intensity RT yields the best outcomes for patients with KOA [50], as it provides adequate muscle stimulation without overburdening the knee joint [51], thereby enhancing muscle strength and endurance, which in turn improves the mechanical stability and function of the joint [52]. This balance ensures that the therapeutic effects are maximized, making moderate-intensity RT a highly effective intervention for managing KOA.

Interestingly, our analysis revealed that the optimal period and repetition frequency for alleviating pain and improving function in patients with KOA are quite similar. The optimal period for pain reduction was identified as 35 weeks with a weekly repetition frequency of 640, while for functional improvement, the optimal period was 37 weeks with 610 repetitions per week. However, the optimal period and frequency for reducing stiffness were different, with the best outcomes observed at 12 weeks and 1200 repetitions per week. This indicates that reducing stiffness requires a higher frequency of RT over a shorter period, whereas alleviating pain and improving function benefit from a longer duration with moderate frequency. These differences likely stem from the distinct pathological mechanisms and muscle responses associated with each symptom. Improving pain and function may necessitate prolonged and progressive muscle strengthening and adaptation [53], while reducing stiffness might require intense, short-term stimulation to rapidly enhance muscle and joint flexibility [54]. These findings provide crucial insights for designing individualized RT programs targeting specific symptoms in patients with KOA. By tailoring the duration and frequency of RT to address the particular needs of pain, stiffness, and functional impairment, clinicians can significantly enhance the overall treatment efficacy for KOA.

This study’s primary strength lies in its comprehensive and systematic approach. By rigorously adhering to the PRISMA guidelines and employing a network meta-analysis, we were able to integrate and compare data from multiple high-quality studies, providing reliable conclusions about the effectiveness of various RT regimens for patients with KOA. Additionally, the strict inclusion criteria, which only encompassed RCTs, significantly enhanced the internal validity of our findings and reduced the risk of bias. However, several limitations must be considered. First, despite our exhaustive search and selection process, there is inherent variability in the RT protocols used across the included studies. Differences in training intensity, frequency, duration, and specific exercises may introduce heterogeneity that could affect the generalizability of our results. Second, although these outcomes are clinically relevant and commonly used in KOA research, the reliance on self-reported measures of pain, stiffness, and function could introduce subjectivity and measurement bias. Third, while we attempted to control for potential confounding variables through rigorous study design and analysis, residual confounding cannot be entirely ruled out, particularly given the complexity of KOA and the multifactorial nature of its symptom. Furthermore, as some interventions were based on a relatively small number of studies and participants, these findings should be interpreted with caution. Further high-quality randomized controlled trials are needed to further evaluate these effects.

## Conclusions

This study demonstrates that high-speed RT is the most effective method for alleviating pain, reducing stiffness, and enhancing function in patients with KOA. Specifically, moderate-intensity RT (43–47% 1RM) for 35–37 weeks, with 610–640 repetitions per week, was found to be optimal for reducing pain and enhancing function. For stiffness, a higher frequency and shorter duration (12 weeks, 1200 repetitions per week) were more effective. These results highlight the necessity of personalized RT programs tailored to patients with KOA to maximize therapeutic outcomes and provide crucial insights for clinical guidelines and individualized patient care. Future research should standardize RT protocols and employ objective measures to further validate these findings.

## Supplementary Information

Supplementary Material 1.

## Data Availability

Extracted data and analysis codes are available from Yong Yang upon reasonable request.

## Abbreviations

95% CrI: 95% Credible Interval
CON: Control group
KOA: Knee Osteoarthritis
RCT: Randomized controlled trials
RT: Resistance training
SD: Standard Deviation
SMD: Standardized Mean Difference
