# Supplementary material for: Optimal resistance training strategies for knee osteoarthritis symptom relief: a systematic review and network meta-analysis
Source: BMC Musculoskelet Disord. 2025 Dec 12;27:48. doi: 10.1186/s12891-025-09341-0 (PMC12821314; doi:10.1186/s12891-025-09341-0)
Supplement: Supplementary file 1 — Supplementary Material 1. [file 12891_2025_9341_MOESM1_ESM.docx]

**Supplementary**

Table of Contents:

[Supplementary 1: Search Strategy 3](#_Toc195051508)

[1.1 Database: PubMed <inception to April 23, 2025> 3](#_Toc195051509)

[1.2 Database: Ovid MEDLINE(R) < inception to April 23, 2025 4](#_Toc195051510)

[1.3 Database: Embase <1974 to April 23, 2025> 5](#_Toc195051511)

[1.4 Database: Web of Science <1965 to April 23, 2025> 6](#_Toc195051512)

[1.5 Cochrane 7](#_Toc195051513)

[Supplementary 2: Definitions of resistance traing types and non-exercise training control 8](#_Toc195051514)

[Supplementary 3: Assessment of the transitivity 9](#_Toc195051515)

[3.1 Publish years 9](#_Toc195051516)

[3.2 Mean age 10](#_Toc195051517)

[3.3 Percentage male 11](#_Toc195051518)

[3.4 Sample size 12](#_Toc195051519)

[3.5 Disease grade 13](#_Toc195051520)

[Supplementary 4: Characteristics of resistance training included in the review 14](#_Toc195051521)

[List of included literature 20](#_Toc195051522)

[Supplementary 5: Risk of Bias 24](#_Toc195051523)

[Table 5.1 The risk of bias assessment for the individual included studies 24](#_Toc195051524)

[Supplementary 6: Evaluation of heterogeneity and inconsistency 27](#_Toc195051525)

[Supplementary 7: Network plot 35](#_Toc195051526)

[Supplementary 8: League table 38](#_Toc195051527)

[Supplementary 9: Publication bias 41](#_Toc195051528)

[Supplementary 10: Grading the evidence of the network meta-analysis using CINeMA 44](#_Toc195051529)

[10.1 CINeMA for Pain 44](#_Toc195051530)

[10.2 CINeMA for Stiffness 47](#_Toc195051531)

[10.3 CINeMA for Function 50](#_Toc195051532)

[Supplementary 11: Assessment of Connectivity, Consistency and Transitivity in Network Meta Dose-Response Analysis 53](#_Toc195051533)

[Supplementary 12: Non-linear functions and models fit comparison 75](#_Toc195051534)

# Supplementary 1: Search Strategy

## 1.1 Database: PubMed <inception to April 2, 2025>

| #15 | (((osteoarthritis, knee[MeSH Terms]) OR ("knee osteoarthritis" OR "osteoarthritis of knee" OR "osteoarthritis of the knee")) AND (((exercise[MeSH Terms]) OR (resistance training[MeSH Terms])) OR ("strength training" OR "weight training" OR "power training" OR "plyometric training" OR "complex training" OR "weight-bearing exercise"))) AND ((((((randomized controlled trial[Publication Type]) OR (controlled clinical trial[Publication Type])) OR (randomized[Title/Abstract])) OR (placebo[Title/Abstract])) OR (randomly[Title/Abstract])) OR (trial[Title])) |
| --- | --- |
| #14 | (((((randomized controlled trial[Publication Type]) OR (controlled clinical trial[Publication Type])) OR (randomized[Title/Abstract])) OR (placebo[Title/Abstract])) OR (randomly[Title/Abstract])) OR (trial[Title]) |
| #13 | ((exercise[MeSH Terms]) OR (resistance training[MeSH Terms])) OR ("strength training" OR "weight training" OR "power training" OR "plyometric training" OR "complex training" OR "weight-bearing exercise") |
| #12 | (osteoarthritis, knee[MeSH Terms]) OR ("knee osteoarthritis" OR "osteoarthritis of knee" OR "osteoarthritis of the knee") |
| #11 | trial[Title] |
| #10 | randomly[Title/Abstract] |
| #9 | placebo[Title/Abstract] |
| #8 | randomized[Title/Abstract] |
| #7 | controlled clinical trial[Publication Type] |
| #6 | randomized controlled trial[Publication Type] |
| #5 | "strength training" OR "weight training" OR "power training" OR "plyometric training" OR "complex training" OR "weight-bearing exercise" |
| #4 | resistance training[MeSH Terms] |
| #3 | exercise[MeSH Terms] |
| #2 | "knee osteoarthritis" OR "osteoarthritis of knee" OR "osteoarthritis of the knee" |
| #1 | osteoarthritis, knee[MeSH Terms] |

## 1.2 Database: Ovid MEDLINE(R) < inception to April 2, 2025

| #1 | exp Osteoarthritis, Knee/ |
| --- | --- |
| #2 | (' osteoarthritis, knee' or 'osteoarthritis of knee' or 'osteoarthritis of the knee').mp. [mp=title, abstract, original title, name of substance word, subject heading word, floating sub-heading word, keyword heading word, organism supplementary concept word, protocol supplementary concept word, rare disease supplementary concept word, unique identifier, synonyms] |
| #3 | exp Resistance Training/ |
| #4 | ('strength training' or 'weight training' or 'power training' or 'plyometric training' or 'complex training' or 'weight-bearing exercise').mp. [mp=title, abstract, original title, name of substance word, subject heading word, floating sub-heading word, keyword heading word, organism supplementary concept word, protocol supplementary concept word, rare disease supplementary concept word, unique identifier, synonyms] |
| #5 | exp Randomized Controlled Trial/ |
| #6 | 'clinical trial'.mp. |
| #7 | trial.m_titl. |
| #8 | randomly.mp. |
| #9 | randomized.mp. |
| #10 | 1 or 2 |
| #11 | 3 or 4 |
| #12 | 5 or 6 or 7 or 8 or 9 |
| #13 | 10 and 11 and 12 |

## 1.3 Database: Embase <1974 to April 2, 2025>

| #13 | #10 AND #11 AND #12 |
| --- | --- |
| #12 | #3 OR #4 |
| #11 | #1 OR #2 |
| #10 | #5 OR #6 OR #7 OR #8 OR #9 |
| #9 | 'randomized controlled trials'/exp |
| #8 | 'clinical trial'/exp |
| #7 | trial:ti |
| #6 | randomly:ab |
| #5 | randomized:ab |
| #4 | 'strength training' OR 'weight training' OR 'power training' OR 'plyometric training' OR 'complex training' OR 'weight-bearing exercise' |
| #3 | 'resistance training'/exp |
| #2 | ' osteoarthritis, knee' OR 'osteoarthritis of knee' OR 'osteoarthritis of the knee' |
| #1 | 'knee osteoarthritis'/exp |

## 1.4 Database: Web of Science <1965 to April 2, 2025>

| #4 | #3 AND #2 AND #1 |
| --- | --- |
| #3 | TOPIC: ((((((((("randomized controlled trial*") OR "controlled clinical trial") OR "random*") OR "clinical trial*") OR randomly) OR trial) OR "clinical trial") OR "randomized controlled trial*") OR "cross-over studies") OR clinic*)  Indexes=SCI-EXPANDED, SSCI, A&HCI, CPCI-S, CPCI-SSH, BKCI-S, BKCI-SSH, ESCI, CCR-EXPANDED, IC Timespan=All years |
| #2 | TOPIC:(((((('resistance) training' OR 'strength training') OR 'weight training') OR 'power training') OR 'plyometric training') OR 'complex training') OR 'weight-bearing exercise')  Indexes=SCI-EXPANDED, SSCI, A&HCI, CPCI-S, CPCI-SSH, BKCI-S, BKCI-SSH, ESCI, CCR-EXPANDED, IC Timespan=All years |
| #1 | TOPIC:  ((('osteoarthritis,) knee' OR 'osteoarthritis of knee') OR 'osteoarthritis of the knee') OR 'knee osteoarthritis')  Indexes=SCI-EXPANDED, SSCI, A&HCI, CPCI-S, CPCI-SSH, BKCI-S,  BKCI-SSH, ESCI, CCR-EXPANDED, IC Timespan=All years |

## 1.5 Cochrane

| #1 | MeSH descriptor: [Osteoarthritis, Knee] explode all trees |
| --- | --- |
| #2 | ' osteoarthritis, knee' OR 'osteoarthritis of knee' OR 'osteoarthritis of the knee' |
| #3 | MeSH descriptor: [Resistance Training] explode all trees |
| #4 | MeSH descriptor: [exercise] explode all trees |
| #5 | 'strength training' OR 'weight training' OR 'power training' OR 'plyometric training' OR 'complex training' OR 'weight-bearing exercise' |
| #6 | #1 or #2 |
| #7 | #3 or #4 or #5 |
| #8 | #6 and #7 |

# Supplementary 2: Definitions of resistance traing types and non-exercise training control

| **Abbreviation** | **Full name** | **Definitions** |
| --- | --- | --- |
| ACCEIT | Aquatic resistance training, Combined Concentric-Eccentric Isotonic Training | Resistance training performed in water, muscle contraction patterns include concentric, isometric and eccentric isotonic contractions. |
| CCEIT | Combined Concentric-Eccentric Isotonic Training | Traditional resistance training, muscle contraction patterns include concentric, isometric and eccentric isotonic contractions. |
| CIT | Concentric Isotonic Training | During resistance training, perform only concentric isotonic contractions. |
| CON | Control group | Non-exercise intervention, usual care [1], or health education |
| EIT | Eccentric Isotonic Training | During resistance training, perform only eccentric isotonic contractions. |
| High speed | Combined Concentric-Eccentric Isotonic Training, as fast as possible | During resistance training, complete the movements as quickly as possible. |
| Isokinetic | Combined Concentric-Eccentric Isokinetic Training | Resistance training at a constant speed of movement. |
| Isometric | Isometric muscle contraction | During resistance training, perform only isometric contractions. |

# Supplementary 3: Assessment of the transitivity

Different clinical trials need to ensure that their baseline levels are consistent. If the baseline levels are inconsistent, the results cannot be transitive. Therefore, the transitivity assumption was evaluated by comparing the distribution of potential effect modifiers (publication year, sample size, mean age, percentage male, disease grade [2] across studies grouped before analyzing the results, and we use the R ggplot2 package to draw boxplots between the above potential influencing factors and various types of resistance training.

## 3.1 Publish years

We checked the publication year distribution of the included studies. The range is from 1996 to 2022, with a median of 2013. The results of one-way ANOVA showed that there was statistical difference in the years of publication between the types of resistance training (F = 1.994, P = 0.0627).

******

**Figure 3.1:** Boxplot for distribution of publication year. ACCEIT Combined Concentric-Eccentric Isotonic Training in water; CCEIT Combined Concentric-Eccentric Isotonic Training; CIT Concentric Isotonic Training; CON Control group; EIT Eccentric Isotonic Training; High speed Combined Concentric-Eccentric Isotonic Training, as fast as possible; Isokinetic Combined Concentric-Eccentric Isokinetic Training; Isometric Isometric muscle contraction.

## 3.2 Mean age

We checked the mean age distribution of the included study participants. The range is from 52.8 to 74.5, with a median of 64. The results of one-way ANOVA showed that there was no statistical difference in the mean age between the types of resistance training (F = 1.219, P = 0.3).

******

**Figure 3.2:** Boxplot for distribution of mean age. ACCEIT Combined Concentric-Eccentric Isotonic Training in water; CCEIT Combined Concentric-Eccentric Isotonic Training; CIT Concentric Isotonic Training; CON Control group; EIT Eccentric Isotonic Training; High speed Combined Concentric-Eccentric Isotonic Training, as fast as possible; Isokinetic Combined Concentric-Eccentric Isokinetic Training; Isometric Isometric muscle contraction.

## 3.3 Percentage male

We checked the percentage male distribution of the included study participants. The range is from 0% to 61.3%, with a median of 25%. The results of one-way ANOVA showed that there was no statistical difference in the percentage male between the types of resistance training (F = 1.242, P = 0.29).

******

**Figure 3.3:** Boxplot for distribution of percentage male. ACCEIT Combined Concentric-Eccentric Isotonic Training in water; CCEIT Combined Concentric-Eccentric Isotonic Training; CIT Concentric Isotonic Training; CON Control group; EIT Eccentric Isotonic Training; High speed Combined Concentric-Eccentric Isotonic Training, as fast as possible; Isokinetic Combined Concentric-Eccentric Isokinetic Training; Isometric Isometric muscle contraction.

## 3.4 Sample size

We checked the sample size distribution of the included studies each arm. The range is from 6 to 128, with a median of 30. The results of one-way ANOVA showed that there was no statistical difference in the sample size between the types of resistance training (F = 0.697, P = 0.674).

**Figure 3.4:** Boxplot for distribution of sample size. ACCEIT Combined Concentric-Eccentric Isotonic Training in water; CCEIT Combined Concentric-Eccentric Isotonic Training; CIT Concentric Isotonic Training; CON Control group; EIT Eccentric Isotonic Training; High speed Combined Concentric-Eccentric Isotonic Training, as fast as possible; Isokinetic Combined Concentric-Eccentric Isokinetic Training; Isometric Isometric muscle contraction.

## 3.5 Disease grade

We checked the disease grade (K/L grade) distribution of the included studies each arm. The range is from 1.37 to 3.5, with a median of 2.14. The results of one-way ANOVA showed that there was no statistical difference in the disease grade between the types of resistance training (F = 1.632, P = 0.187).

**Figure 3.5:** Boxplot for distribution of disease grade. ACCEIT Combined Concentric-Eccentric Isotonic Training in water; CCEIT Combined Concentric-Eccentric Isotonic Training; CIT Concentric Isotonic Training; CON Control group; EIT Eccentric Isotonic Training; High speed Combined Concentric-Eccentric Isotonic Training, as fast as possible; Isokinetic Combined Concentric-Eccentric Isokinetic Training; Isometric Isometric muscle contraction.

# Supplementary 4: Characteristics of resistance training included in the review

| **Study** | **Training dose** | **Period (week)** | **Frequency (times/week)** | **Source of resistance** | **Training exercises** | **Target muscles** |
| --- | --- | --- | --- | --- | --- | --- |
| Oliveira, et al. (2012) [1] | 50%-60% 1RM; Sets:3; Reps: 15; Rest(s): 30-40/set | 8 | 3 | Based on elastic band | Knee extension | Quadriceps |
| de Almeida, et al. (2020) [2] | RT1: 6-20 RPE; Reps: NA; Sets: 1; Rest(s): 30/exercise RT2: 25-50% 1RM; Sets: 2; Reps:15 | 14 | 3 | Based on self-weight | RT1: 15-17 exercises, full body resistance training RT2: Knee flexion and extension, hip adduction and abduction, | RT1: upper and lower limbs and trunk RT2: quadriceps and hamstrings |
| de Almeida, et al. (2021) [3] | RT1: 6-20 RPE; Reps: NA; Sets: 1; Rest(s): 30/exercise RT2: 25-50% 1RM; Sets: 2; Reps:15 | 14 | 3 | Based on self-weight | RT1: 15-17 exercises, full body resistance training RT2: Knee flexion and extension, hip adduction and abduction | RT1: upper and lower limbs and trunk RT2: quadriceps and hamstrings |
| Waller, et al. (2017) [4] | RPE 13-14; Reps: NA; Sets: 1; Rest(s): 30/exercise | 16 | 3 | Based on water | 14 exercises, full body resistance training | Upper and lower limbs and trunk |
| Lim, et al. (2008) [5] | high intensity using black elastic band; Sets: 2-3; Reps: 10 | 12 | 5 | Based on elastic band | 5 exercises, knee extension | Quadriceps |
| Gür, et al. (2002) [6] | high intensity; Reps: 12; Sets: 1; Rest(s): 120 | 8 | 3 | Based on machine | RT1: 6 concentric extension, eccentric extension movements, then 6 concentric flexion, eccentric flexion movements RT2: 12 concentric extension, concentric flexion movements | Quadriceps and hamstrings |
| Farr, et al. (2010) [7] | 50%-75% 3RM; Sets:1-2; Reps: 6-8; | 36 | 3 | Based on elastic band | Leg press, Incline dumbbell press, seated row, Leg curl, Calf raise | Upper and lower limbs |
| Vincent, et al. (2019) [8] | RT1: RPE 17-18 of 20 point, 60-100% 1RM; Sets: 1; Reps: 8 RT2: RPE 17-18 of 20 points, 60% 1RM; Sets: 1; Reps: 12 | 16 | 2 | Based on machine | Leg press, knee flexion, knee extension, chest press, seated row, overhead press, biceps curl, and calf press. | Upper and lower limbs |
| Pazit, et al. (2018) [9] | 20-80% 1RM; Sets: 2-3, Reps: 2-12 | 8 | 2 | Based on self-weight | Leg press, sit to stand, squat, step-up, calf raises lunges, going up stairs | Lower limbs |
| Rogers, et al. (2012) [10] | 65%1RM; Sets:1; Reps: 15 | 8 | 3 | Based on elastic band | Seated: Ankle extension, ankle flexion, knee extension, knee flexion, hip abduction, hip adduction, hip internal rotation, hip external rotation, leg press (hip and knee extension) Standing: Hip hyper-extension | Lower limbs |
| Jan, et al. (2008) [11] | RT1: 60%1RM; Sets: 3; Reps: 8 RT2: 10% 1RM; Sets: 10; Reps: 15 | 8 | 3 | Based on machine | Knee flexion and extension | Quadriceps and hamstrings |
| Wortley, et al. (2013) [12] | RT based on ankle weight   Sets:2-3; Reps: 8-12 | 10 | 2 | Based on self-weight | seated leg extension, standing hamstring curl, straight leg raise, standing hip abduction, standing hip adduction, standing hip flexion, standing calf raise | Quadriceps and hamstrings |
| Foroughi, et al. (2011) [13] | RPE: 15-18; Sets: 3; Reps: 8; Rest(s): 60-120 | 24 | 3 | Based on machine | Unilateral knee extension, standing hip abduction and adduction; and bilateral knee flexion, leg press, and plantar-flexion | Lower limbs |
| Foroughi, et al. (2011) [14] | RPE: 15-18; Sets: 3; Reps: 8; Rest(s): 60-120 | 24 | 3 | Based on machine | Unilateral knee extension, standing hip abduction and adduction; and bilateral knee flexion, leg press, and plantar-flexion | Lower limbs |
| DeVita, et al. (2018) [15] | 55%–80% 1RM; Sets:3; Reps: 10 | 12 | 3 | Based on machine | leg extension, leg press and forward lunge exercises | Quadriceps |
| Jorge, et al. (2015) [16] | 50%-70% 1RM; Sets:2; Reps: 8; Rest(s):60 | 12 | 2 | Based on machine | knee EXtension/flexion and hip abduction/adduction using two gym machines (knee flexion-extension and abduction- exadduction) with free weights | Quadriceps and hamstrings |
| Bruce-Brand, et al. (2012) [17] | RPE: ≥14 (15 point Borg scale); Sets:3; Reps: 10 | 8 | 3 | Based on elastic band | knee presses, bottle knee presses, extended leg raises, leg extensions, wall squats and hamstring curls | Lower limbs |
| Topp, et al. (2002) [18] | RT1: 11-12 RPE, Sets: 1-3, Reps: 8-12, Rest(s): 120 RT2: 11-12 RPE, Sets: 1-3, Reps: 8-12, Rest(s): 120, maximum muscle tension: 3-5s | 16 | 3 | Based on elastic band | RT1: 6 exercises, ankle dorsi- and plantarflexors, knee flexors and extensors, and hip flexion and extension RT2: 6 exercises, 0° of dorsi- and plantarflexion, 10° of knee flexion and RTtension, 10° of hip flexion and 10° of hip extension | Lower limbs |
| Isaramalai, et al. (2018) [19] | 50%-100% 10RM; Sets:3; Reps: 10, 5 seconds holds | 8 | 3 | RT1: Based on self-weight RT2: Based on Sandbags | straight-leg raise (sitting), hip abduction (standing), Hip flexion (standing), hip extension (standing), Knee flexion (standing), Knee flexion (lying prone), hip extension (lying prone), straight-leg raise (supine), Quad set | Lower limbs |
| Oh, et al. (2021) [20] | low-resistance yellow loop bands; Sets: 1; Reps(s): 15-20 | 20 | 2-3 | Based on elastic band | 12 exercises | Lower limbs |
| Assar, et al. (2020) [21] | RPE 11-15; Sets:3; Reps: 10-15; | 8 | 3 | Based on TRX | 9 exercises | RT1: core muscles, hip abductors, and leg muscles strengthening |
| Messier, et al. (2021) [22] | RT1: 75%-90%1RM, Sets: 3, Reps: 4-8 RT2: 30%-40%1RM; Sets:3; Reps: 15 | 72 | 3 | Based on machine | hip abduction and adduction; leg curl, leg extension, and leg press; and seated cal, compound row, vertical chest, lower back, and abdomen. | Upper and lower limbs and trunk |
| Chang, et al. (2012) [23] | RPE: 13; Sets: 3; Reps: 10; Rest(s): 60-120 | 8 | 2-3 | Based on elastic band | Leg press | Quadriceps and hamstrings |
| Baker, et al. (2001) [24] | 6 (RPE0-10); Sets: 2; Reps: 10 | 16 | 3 | Based on machine | Hip flexion/extension, hip abduction/adduction, knee extension/flexion | Quadriceps and hamstrings |
| Salli, et al. (2010) [25] | RT1: 70% 1RM, Sets: 5, 60°/s to 180°/s; Reps:10 RT2: 70% 1RM, Sets: 5; Reps: 10, 5 seconds holds | 8 | 3 | Based on machine | knee extension/flexion | Quadriceps and hamstrings |
| Lin, et al. (2009) [26] | 50%-70%1RM ; Sets:4; Reps: 6; Rest(s):60 | 8 | 3 | Based on self-weight | knee extension/flexion | Quadriceps and hamstrings |
| Jan, et al. (2009) [27] | 50%-70%1RM; Sets:4; Reps: 6; Rest(s):60 | 8 | 3 | RT1: Based on machine RT2: Based on self-weight | RT1: leg press RT2: knee extension/flexion | Quadriceps and hamstrings |
| Bennell, et al. (2010) [28] | RT based on ankle cuff weights or elastic bands Intensity:10RM; Sets:3; Reps: 10, or 5 seconds holds | 12 | 5 | Based on ankle cuff weights or elastic bands | Hip abduction in sidelying, hip abduction in standing, Standing wall isometric hip abduction, Hip adduction in sidelying, Hip adduction in standing, Bilateral isometric hip adduction | Hip adduction and abduction muscle groups |
| O'Reilly, et al. (1999) [29] | 55% 1RM; Sets: 1; Reps: 20 | 24 | 7 | Based on self-weight, and elastic band | Isometric quadriceps contraction, Isotonic quadriceps contraction, Isotonic hamstring contraction, Isotonic quadriceps contraction with resistance band held for five seconds | Quadriceps and hamstrings |
| Schilke, et al. (1996) [30] | RT based on mechine 50-75% 1RM, 90°/s, Sets: 6; Reps: 5 | 8 | 3 | Based on machine | knee extension/flexion | Quadriceps and hamstrings |
| Rafiq, et al. (2021) [31] | RPE: 10-11; Sets: 2; Reps: 7-10 | 4 | 3 | Based on self-weight, and elastic band | hip abduction and adduction, Hip flexion, and extension, knee extension, and flexion, Isotonic hamstring contraction, Ankle dorsiflexors, Ankle plantar flexors | Lower limbs |
| Foley, et al. (2003) [32] | 75% 1RM, Sets:3; Reps: 10-15 | 6 | 3 | RT1: Based on water RT2: Based on machine | RT1: hip flexion and extension, hip adduction and abduction, knee flexion and extension, and knee cycling RT2: seated bench press, hip adduction and abduction, knee extension, and double leg press | RT1: quadriceps and hamstrings RT2: upper and lower limbs and trunk |
| Chen, et al. (2019) [33] | RT1: RPE 13; Reps: 10, Sets: 5 RT2: RPE 13; Reps: 10, 10s hold; Sets: 5 | 12 | 3 | Based on elastic band | Hip joint abduction/adduction, flexion/extension, external/internal rotation, knee joint flexion/extension, and ankle joint plantar/dorsiflexion | Lower limbs |
| Evcik, et al. (2002) [34] | 0.5 kg to 5 kg load; Sets: 1; Reps: 10, 5s hold | 12 | 14 | Based on machine | Isometric straight leg lifts, Isometric quadriceps contraction | Quadriceps |
| Vassão, et al. (2021) [35] | 60%1RM; Sets:3; Reps:8; Rest(s):120-180 | 8 | 2 | Based on self-weight | Hip abductors and adductors chair, seated leg raise, Glute Bridge (Hip lift), Knee flexors and extensors chair | Quadriceps and hamstrings |
| Munukka, et al. (2020) [36] | RT based on mechine in the water Intensity:PRE12-18; Sets:2-3; Reps: 12-18; Rest(s):30-45 | 16 | 3 | Based on water | Standing hip flexion/extension, Standing hip abduction/adduction, Seated bilateral knee flexion/extension, Standing knee flexion/extension, Calf raises on edge of step, Standing abdominals, scissor jumps | Upper and lower limbs and trunk |
| Silva, et al. (2008) [37] | RT1: 20-30% 1RM; Reps: 7-10, 6s hold; Sets: 1 RT1: 20-30% 1RM; Reps: 20-40; Sets: 1 | 18 | 3 | RT1: Based on water RT2: Based on self-weight | knees straight, knee flexion, knee extension, Hip abduction, Hip adduction | Quadriceps and hamstrings |
| Chen, et al. (2014) [38] | 60% 1RM, 30-120°/s; Sets:1-5; Reps:5; Rest(s):5 | 8 | 3 | Based on machine | Knee flexion and extension | Quadriceps and hamstrings |
| Huang, et al. (2003) [39] | RT1: 60% 1RM, 30-120°/s; Sets:1-5; Reps:5; Rest(s):5 RT2: 60% 1RM; Sets:1-5; Reps:5; Rest(s):5 RT3: 60% 1RM, hold 5s; Sets:1-5; Reps:5; Rest(s):5 | 8 | 3 | Based on machine | Knee flexion and extension | Quadriceps and hamstrings |
| Huang, et al. (2005) [40] | 60-80% 1RM, 30-120°/s; Sets:1-5; Reps:5; Rest(s):5 | 8 | 3 | Based on machine | Knee flexion and extension | Quadriceps and hamstrings |
| Huang, et al. (2018) [41] | hold 10s; Reps: 10; Sets: 1 | 4 | 3 | Based on self-weight | knees straight, Hip abduction, Hip adduction | Lower limbs |
| Munukka, et al. (2016) [42] | RT based on mechine in the water Intensity:PRE12-18; Sets:2-3; Reps: 12-18; Rest(s):30-45 | 16 | 3 | Based on water | Standing hip flexion/extension, Standing hip abduction/adduction, Seated bilateral knee flexion/extension, Standing knee flexion/extension, Calf raises on edge of step, Standing abdominals, scissor jumps | Upper and lower limbs and trunk |
| Samut, et al. (2015) [43] | 65-75% 1RM, 60 °/s, 90 °/s, 120 °/s and 180 °/s; Sets: 5; Reps: 5 | 6 | 3 | Based on machine | Knee flexion and extension | Quadriceps and hamstrings |
| Weng, et al. (2009) [44] | 60% 1RM, 30-120°/s; Sets:1-5; Reps:5; Rest(s):5 | 8 | 3 | Based on machine | Knee flexion and extension | Quadriceps and hamstrings |
| Vincent, et al. (2020) [45] | RT1: RPE 17-18 of 20 point, 60-100% 1RM; Sets: 1; Reps: 8 RT2: RPE 17-18 of 20 points, 60% 1RM; Sets: 1; Reps: 12 | 16 | 2 | Based on machine | Leg press, knee flexion, knee extension, chest press, seated row, overhead press, biceps curl, and calf press. | Upper and lower limbs |
| Trojani, et al. (2022) [46] | 60-80% 1RM; Sets: 1-3; Reps: 8-12; Rest(s): 60-180s; | 6 | 2 | Based on machine | Leg press, knee flexion, knee extension, chest press, seated row, overhead press, biceps curl, and calf press. | Upper and lower limbs |

# List of included literature

1. Oliveira AMId, Peccin MS, Silva KNGd, Teixeira LEPdP, Trevisani VFM. Impact of exercise on the functional capacity and pain of patients with knee osteoarthritis: a randomized clinical trial. Rev Bras Reumatol. 2012;52(6):876-882. doi. https://pubmed.ncbi.nlm.nih.gov/23223698.

2. de Almeida AC, Aily JB, Pedroso MG, et al. A periodized training attenuates thigh intermuscular fat and improves muscle quality in patients with knee osteoarthritis: results from a randomized controlled trial. Clin Rheumatol. 2020;39(4):1265-1275. doi: 10.1007/s10067-019-04892-9.

3. de Almeida AC, Aily JB, Pedroso MG, Gonçalves GH, Pastre CM, Mattiello SM. Reductions of cardiovascular and metabolic risk factors after a 14-week periodized training model in patients with knee osteoarthritis: a randomized controlled trial. Clin Rheumatol. 2021;40(1):303-314. doi: 10.1007/s10067-020-05213-1.

4. Waller B, Munukka M, Rantalainen T, et al. Effects of high intensity resistance aquatic training on body composition and walking speed in women with mild knee osteoarthritis: a 4-month RCT with 12-month follow-up. Osteoarthritis Cartilage. 2017;25(8):1238-1246. doi: 10.1016/j.joca.2017.02.800.

5. Lim B-W, Hinman RS, Wrigley TV, Sharma L, Bennell KL. Does knee malalignment mediate the effects of quadriceps strengthening on knee adduction moment, pain, and function in medial knee osteoarthritis? A randomized controlled trial. Arthritis Rheum. 2008;59(7):943-951. doi: 10.1002/art.23823.

6. Gür H, Cakin N, Akova B, Okay E, Küçükoğlu S. Concentric versus combined concentric-eccentric isokinetic training: effects on functional capacity and symptoms in patients with osteoarthrosis of the knee. Arch Phys Med Rehabil. 2002;83(3):308-316. doi. https://pubmed.ncbi.nlm.nih.gov/11887109.

7. Farr JN, Going SB, McKnight PE, Kasle S, Cussler EC, Cornett M. Progressive resistance training improves overall physical activity levels in patients with early osteoarthritis of the knee: a randomized controlled trial. Phys Ther. 2010;90(3):356-366. doi: 10.2522/ptj.20090041.

8. Vincent KR, Vasilopoulos T, Montero C, Vincent HK. Eccentric and Concentric Resistance Exercise Comparison for Knee Osteoarthritis. Med Sci Sports Exerc. 2019;51(10):1977-1986. doi: 10.1249/MSS.0000000000002010.

9. Pazit L, Jeremy D, Nancy B, Michael B, George E, Hill KD. Safety and feasibility of high speed resistance training with and without balance exercises for knee osteoarthritis: A pilot randomised controlled trial. Phys Ther Sport. 2018;34:154-163. doi: 10.1016/j.ptsp.2018.10.001.

10. Rogers MW, Tamulevicius N, Semple SJ, Krkeljas Z. Efficacy of home-based kinesthesia, balance & agility exercise training among persons with symptomatic knee osteoarthritis. J Sports Sci Med. 2012;11(4):751-758. doi. https://pubmed.ncbi.nlm.nih.gov/24150088.

11. Jan M-H, Lin J-J, Liau J-J, Lin Y-F, Lin D-H. Investigation of clinical effects of high- and low-resistance training for patients with knee osteoarthritis: a randomized controlled trial. Phys Ther. 2008;88(4):427-436. doi: 10.2522/ptj.20060300.

12. Wortley M, Zhang S, Paquette M, et al. Effects of resistance and Tai Ji training on mobility and symptoms in knee osteoarthritis patients. Journal of Sport and Health Science. 2013;2(4):209-214. doi.

13. Foroughi N, Smith RM, Lange AK, Baker MK, Fiatarone Singh MA, Vanwanseele B. Lower limb muscle strengthening does not change frontal plane moments in women with knee osteoarthritis: A randomized controlled trial. Clin Biomech (Bristol, Avon). 2011;26(2):167-174. doi: 10.1016/j.clinbiomech.2010.08.011.

14. Foroughi N, Smith RM, Lange AK, Singh MAF, Vanwanseele B. Progressive resistance training and dynamic alignment in osteoarthritis: A single-blind randomised controlled trial. Clin Biomech (Bristol, Avon). 2011;26(1):71-77. doi: 10.1016/j.clinbiomech.2010.08.013.

15. DeVita P, Aaboe J, Bartholdy C, Leonardis JM, Bliddal H, Henriksen M. Quadriceps-strengthening exercise and quadriceps and knee biomechanics during walking in knee osteoarthritis: A two-centre randomized controlled trial. Clin Biomech (Bristol, Avon). 2018;59:199-206. doi: 10.1016/j.clinbiomech.2018.09.016.

16. Jorge RTB, Souza MCd, Chiari A, et al. Progressive resistance exercise in women with osteoarthritis of the knee: a randomized controlled trial. Clin Rehabil. 2015;29(3):234-243. doi: 10.1177/0269215514540920.

17. Bruce-Brand RA, Walls RJ, Ong JC, Emerson BS, O'Byrne JM, Moyna NM. Effects of home-based resistance training and neuromuscular electrical stimulation in knee osteoarthritis: a randomized controlled trial. BMC Musculoskelet Disord. 2012;13:118. doi: 10.1186/1471-2474-13-118.

18. Topp R, Woolley S, Hornyak J, Khuder S, Kahaleh B. The effect of dynamic versus isometric resistance training on pain and functioning among adults with osteoarthritis of the knee. Arch Phys Med Rehabil. 2002;83(9):1187-1195. doi. https://pubmed.ncbi.nlm.nih.gov/12235596.

19. Isaramalai S-A, Hounsri K, Kongkamol C, et al. Integrating participatory ergonomic management in non-weight-bearing exercise and progressive resistance exercise on self-care and functional ability in aged farmers with knee osteoarthritis: a clustered randomized controlled trial. Clin Interv Aging. 2018;13:101-108. doi: 10.2147/CIA.S144288.

20. Oh S-L, Kim D-Y, Bae J-H, Lim J-Y. Effects of rural community-based integrated exercise and health education programs on the mobility function of older adults with knee osteoarthritis. Aging Clin Exp Res. 2021;33(11):3005-3014. doi: 10.1007/s40520-020-01474-7.

21. Assar S, Gandomi F, Mozafari M, Sohaili F. The effect of Total resistance exercise vs. aquatic training on self-reported knee instability, pain, and stiffness in women with knee osteoarthritis: a randomized controlled trial. BMC Sports Sci Med Rehabil. 2020;12:27. doi: 10.1186/s13102-020-00175-y.

22. Messier SP, Mihalko SL, Beavers DP, et al. Effect of High-Intensity Strength Training on Knee Pain and Knee Joint Compressive Forces Among Adults With Knee Osteoarthritis: The START Randomized Clinical Trial. JAMA. 2021;325(7):646-657. doi: 10.1001/jama.2021.0411.

23. Chang T-F, Liou T-H, Chen C-H, Huang Y-C, Chang K-H. Effects of elastic-band exercise on lower-extremity function among female patients with osteoarthritis of the knee. Disabil Rehabil. 2012;34(20):1727-1735. doi: 10.3109/09638288.2012.660598.

24. Baker KR, Nelson ME, Felson DT, Layne JE, Sarno R, Roubenoff R. The efficacy of home based progressive strength training in older adults with knee osteoarthritis: a randomized controlled trial. J Rheumatol. 2001;28(7):1655-1665. doi. https://pubmed.ncbi.nlm.nih.gov/11469475.

25. Salli A, Sahin N, Baskent A, Ugurlu H. The effect of two exercise programs on various functional outcome measures in patients with osteoarthritis of the knee: a randomized controlled clinical trial. Isokinetics and Exercise Science. 2010;18(4):201-209. doi.

26. Lin D-H, Lin C-HJ, Lin Y-F, Jan M-H. Efficacy of 2 non-weight-bearing interventions, proprioception training versus strength training, for patients with knee osteoarthritis: a randomized clinical trial. J Orthop Sports Phys Ther. 2009;39(6):450-457. doi: 10.2519/jospt.2009.2923.

27. Jan M-H, Lin C-H, Lin Y-F, Lin J-J, Lin D-H. Effects of weight-bearing versus nonweight-bearing exercise on function, walking speed, and position sense in participants with knee osteoarthritis: a randomized controlled trial. Arch Phys Med Rehabil. 2009;90(6):897-904. doi: 10.1016/j.apmr.2008.11.018.

28. Bennell KL, Hunt MA, Wrigley TV, et al. Hip strengthening reduces symptoms but not knee load in people with medial knee osteoarthritis and varus malalignment: a randomised controlled trial. Osteoarthritis Cartilage. 2010;18(5):621-628. doi: 10.1016/j.joca.2010.01.010.

29. O'Reilly SC, Muir KR, Doherty M. Effectiveness of home exercise on pain and disability from osteoarthritis of the knee: a randomised controlled trial. Ann Rheum Dis. 1999;58(1):15-19. doi. https://pubmed.ncbi.nlm.nih.gov/10343535.

30. Schilke JM, Johnson GO, Housh TJ, O'Dell JR. Effects of muscle-strength training on the functional status of patients with osteoarthritis of the knee joint. Nurs Res. 1996;45(2):68-72. doi. https://pubmed.ncbi.nlm.nih.gov/8604366.

31. Rafiq MT, Hamid MSA, Hafiz E. Short-Term Effects of Strengthening Exercises of the Lower Limb Rehabilitation Protocol on Pain, Stiffness, Physical Function, and Body Mass Index among Knee Osteoarthritis Participants Who Were Overweight or Obese: A Clinical Trial. ScientificWorldJournal. 2021;2021:6672274. doi: 10.1155/2021/6672274.

32. Foley A, Halbert J, Hewitt T, Crotty M. Does hydrotherapy improve strength and physical function in patients with osteoarthritis--a randomised controlled trial comparing a gym based and a hydrotherapy based strengthening programme. Ann Rheum Dis. 2003;62(12):1162-1167. doi. https://pubmed.ncbi.nlm.nih.gov/14644853.

33. Chen S-M, Shen F-C, Chen J-F, Chang W-D, Chang N-J. Effects of Resistance Exercise on Glycated Hemoglobin and Functional Performance in Older Patients with Comorbid Diabetes Mellitus and Knee Osteoarthritis: A Randomized Trial. Int J Environ Res Public Health. 2019;17(1). doi: 10.3390/ijerph17010224.

34. Evcik D, Sonel B. Effectiveness of a home-based exercise therapy and walking program on osteoarthritis of the knee. Rheumatol Int. 2002;22(3):103-106. doi. https://pubmed.ncbi.nlm.nih.gov/12111084.

35. Vassão PG, de Souza ACF, da Silveira Campos RM, Garcia LA, Tucci HT, Renno ACM. Effects of photobiomodulation and a physical exercise program on the expression of inflammatory and cartilage degradation biomarkers and functional capacity in women with knee osteoarthritis: a randomized blinded study. Adv Rheumatol. 2021;61(1):62. doi: 10.1186/s42358-021-00220-5.

36. Munukka M, Waller B, Häkkinen A, et al. Effects of progressive aquatic resistance training on symptoms and quality of life in women with knee osteoarthritis: A secondary analysis. Scand J Med Sci Sports. 2020;30(6):1064-1072. doi: 10.1111/sms.13630.

37. Silva LE, Valim V, Pessanha APC, et al. Hydrotherapy versus conventional land-based exercise for the management of patients with osteoarthritis of the knee: a randomized clinical trial. Phys Ther. 2008;88(1):12-21. doi. https://pubmed.ncbi.nlm.nih.gov/17986497.

38. Chen T-W, Lin C-W, Lee C-L, et al. The efficacy of shock wave therapy in patients with knee osteoarthritis and popliteal cyamella. Kaohsiung J Med Sci. 2014;30(7):362-370. doi: 10.1016/j.kjms.2014.03.006.

39. Huang M-H, Lin Y-S, Yang R-C, Lee C-L. A comparison of various therapeutic exercises on the functional status of patients with knee osteoarthritis. Semin Arthritis Rheum. 2003;32(6):398-406. doi. https://pubmed.ncbi.nlm.nih.gov/12833248.

40. Huang M-H, Yang R-C, Lee C-L, Chen T-W, Wang M-C. Preliminary results of integrated therapy for patients with knee osteoarthritis. Arthritis Rheum. 2005;53(6):812-820. doi. https://pubmed.ncbi.nlm.nih.gov/16342083.

41. Huang L, Guo B, Xu F, Zhao J. Effects of quadriceps functional exercise with isometric contraction in the treatment of knee osteoarthritis. Int J Rheum Dis. 2018;21(5):952-959. doi: 10.1111/1756-185X.13082.

42. Munukka M, Waller B, Rantalainen T, et al. Efficacy of progressive aquatic resistance training for tibiofemoral cartilage in postmenopausal women with mild knee osteoarthritis: a randomised controlled trial. Osteoarthritis Cartilage. 2016;24(10):1708-1717. doi: 10.1016/j.joca.2016.05.007.

43. Samut G, Dinçer F, Özdemir O. The effect of isokinetic and aerobic exercises on serum interleukin-6 and tumor necrosis factor alpha levels, pain, and functional activity in patients with knee osteoarthritis. Mod Rheumatol. 2015;25(6):919-924. doi: 10.3109/14397595.2015.1038425.

44. Weng M-C, Lee C-L, Chen C-H, et al. Effects of different stretching techniques on the outcomes of isokinetic exercise in patients with knee osteoarthritis. Kaohsiung J Med Sci. 2009;25(6):306-315. doi: 10.1016/S1607-551X(09)70521-2.

45. Vincent KR, Vincent HK. Concentric and Eccentric Resistance Training Comparison on Physical Function and Functional Pain Outcomes in Knee Osteoarthritis: A Randomized Controlled Trial. Am J Phys Med Rehabil. 2020;99(10):932-940. doi: 10.1097/PHM.0000000000001450.

46. Trojani M-C, Chorin F, Gerus P, et al. Concentric or eccentric physical activity for patients with symptomatic osteoarthritis of the knee: a randomized prospective study. Ther Adv Musculoskelet Dis. 2022;14:1759720X221102805. doi: 10.1177/1759720X221102805.

# Supplementary 5: Risk of Bias

## Table 5.1 The risk of bias assessment for the individual included studies

| **Study** | **Randomization process** | **Deviations from intended interventions** | **Mising outcome data** | **Measurement of the outcome** | **Selection of the reported result** | **Overall Bias** |
| --- | --- | --- | --- | --- | --- | --- |
| Oliveira, et al. (2012) [1] | Low | Some concerns | Low | Low | Low | Some concerns |
| de Almeida, et al. (2020) [2] | Low | Low | Low | Low | Low | Low |
| de Almeida, et al. (2021) [3] | Low | Low | Low | Low | Low | Low |
| Waller, et al. (2017) [4] | Some concerns | Low | Low | Low | Some concerns | Some concerns |
| Lim, et al. (2008) [5] | Low | Low | Low | Low | Low | Low |
| Gür, et al. (2002) [6] | Low | Some concerns | Low | Low | Some concerns | Some concerns |
| Farr, et al. (2010) [7] | Low | Some concerns | Some concerns | High | Some concerns | High |
| Vincent, et al. (2019) [8] | Some concerns | Some concerns | Some concerns | Some concerns | Low | Some concerns |
| Pazit, et al. (2018) [9] | Low | Low | Low | Low | Low | Low |
| Rogers, et al. (2012) [10] | Some concerns | Some concerns | Low | Low | Some concerns | Some concerns |
| Jan, et al. (2008) [11] | Low | High | Low | Low | Some concerns | High |
| Wortley, et al. (2013) [12] | Low | High | Some concerns | Some concerns | Some concerns | High |
| Foroughi, et al. (2011) [13] | Low | Some concerns | Some concerns | Some concerns | Some concerns | Some concerns |
| Foroughi, et al. (2011) [14] | Low | Low | Some concerns | Low | Some concerns | Some concerns |
| DeVita, et al. (2018) [15] | Low | Some concerns | High | Low | High | High |
| Jorge, et al. (2015) [16] | Some concerns | Some concerns | Some concerns | Low | Some concerns | Some concerns |
| Bruce-Brand, et al. (2012) [17] | Low | Some concerns | Low | Low | Some concerns | Some concerns |
| Topp, et al. (2002) [18] | Low | Low | Low | Some concerns | Low | Some concerns |
| Isaramalai, et al. (2018) [19] | Some concerns | Low | Some concerns | Some concerns | Some concerns | Some concerns |
| Oh, et al. (2021) [20] | Some concerns | High | Low | Some concerns | Low | High |
| Assar, et al. (2020) [21] | Low | Some concerns | Low | Some concerns | Low | Some concerns |
| Messier, et al. (2021) [22] | Low | Low | Low | Low | Low | Low |
| Chang, et al. (2012) [23] | Low | Some concerns | Some concerns | Some concerns | Some concerns | Some concerns |
| Baker, et al. (2001) [24] | Low | Low | Low | Low | Low | Low |
| Salli, et al. (2010) [25] | Low | Low | Low | Low | Low | Low |
| Lin, et al. (2009) [26] | Low | Some concerns | Some concerns | Some concerns | Some concerns | Some concerns |
| Jan, et al. (2009) [27] | Low | Some concerns | Low | Low | Some concerns | Some concerns |
| Bennell, et al. (2010) [28] | Low | High | Some concerns | High | Some concerns | High |
| O'Reilly, et al. (1999) [29] | Low | Some concerns | Some concerns | Some concerns | Some concerns | Some concerns |
| Schilke, et al. (1996) [30] | Low | Some concerns | Some concerns | Some concerns | Some concerns | Some concerns |
| Rafiq, et al. (2021) [31] | Some concerns | Some concerns | Some concerns | Low | Some concerns | Some concerns |
| Foley, et al. (2003) [32] | Low | High | Low | Some concerns | Some concerns | High |
| Chen, et al. (2019) [33] | Low | Low | Low | Low | Low | Low |
| Evcik, et al. (2002) [34] | Low | Low | Low | Low | Low | Low |
| Vassão, et al. (2021) [35] | Low | Low | Low | Low | Low | Low |
| Munukka, et al. (2020) [36] | Low | Low | Low | Low | Low | Low |
| Silva, et al. (2008) [37] | Low | Low | Low | Low | Low | Low |
| Chen, et al. (2014) [38] | Low | Some concerns | Low | Low | Some concerns | Some concerns |
| Huang, et al. (2003) [39] | Low | Low | Low | Low | Low | Low |
| Huang, et al. (2005) [40] | Low | Low | Low | Low | Low | Low |
| Huang, et al. (2018) [41] | Low | Low | Low | Low | Some concerns | Some concerns |
| Munukka, et al. (2016) [42] | Low | Some concerns | Low | Some concerns | Some concerns | Some concerns |
| Samut, et al. (2015) [43] | Some concerns | Low | Some concerns | Low | Some concerns | Some concerns |
| Weng, et al. (2009) [44] | Low | Some concerns | Low | Some concerns | Some concerns | Some concerns |
| Vincent, et al. (2020) [45] | Low | Low | Low | Low | Low | Low |
| Trojani, et al. (2022) [46] | Low | Low | Low | Low | Low | Low |

# Supplementary 6: Evaluation of heterogeneity and inconsistency

**Table 6.1: Quantifying heterogeneity**

| **Outcomes** | **τ^2^** | **Q** | **df** | **P** | **I^2^** | **Heterogeneity assessment** |
| --- | --- | --- | --- | --- | --- | --- |
| Pain | 0.1207 | 142.80 | 41 | <0.0001 | 64.3% | Moderate to high |
| Stiffness | 0.0240 | 30.19 | 24 | 0.1787 | 20.5% | Low |
| Function | 0.1661 | 124.88 | 37 | <0.0001 | 70.4% | Moderate to high |

**Evaluation of inconsistency**

**Table 6.2 Summary of the global inconsistency and SIDE splitting results**

| Outcomes | the Design-by-Treatment test | | | |
| --- | --- | --- | --- | --- |
|  | Q | df | τ^2^ | p-value |
| Pain | 38.13 | 17 | 0.0710 | 0.2460 |
| Stiffness | 12.09 | 9 | 0.0101 | 0.2085 |
| Function | 14.37 | 11 | 0.1634 | 0.2134 |

**Table 6.3.1 Details of SIDE splitting results (Pain)**

| **Comparison** | **k** | **prop** | **nma** | **direct** | **indir.** | **Diff** | **z** | **p-value** |
| --- | --- | --- | --- | --- | --- | --- | --- | --- |
| ACCEIT vs CCEIT | 1 | 0.22 | 0.001 | -0.4512 | 0.1275 | -0.5787 | -1.21 | 0.2265 |
| ACCEIT vs CIT | 0 | 0 | 0.3 | NA | 0.3 | NA | NA | NA |
| ACCEIT vs CON | 4 | 0.78 | -0.5608 | -0.4 | -1.1337 | 0.7337 | 1.66 | 0.0966 |
| ACCEIT vs EIT | 0 | 0 | 0.3731 | NA | 0.3731 | NA | NA | NA |
| ACCEIT vs High speed | 0 | 0 | 0.7887 | NA | 0.7887 | NA | NA | NA |
| ACCEIT vs Isokinetic | 1 | 0.26 | 0.3059 | -0.1911 | 0.4762 | -0.6674 | -1.34 | 0.1788 |
| ACCEIT vs Isometric | 0 | 0 | 0.2018 | NA | 0.2018 | NA | NA | NA |
| CCEIT vs CIT | 0 | 0 | 0.299 | NA | 0.299 | NA | NA | NA |
| CCEIT vs CON | 25 | 0.96 | -0.5618 | -0.555 | -0.7293 | 0.1743 | 0.38 | 0.7048 |
| CCEIT vs EIT | 0 | 0 | 0.3721 | NA | 0.3721 | NA | NA | NA |
| CCEIT vs High speed | 0 | 0 | 0.7878 | NA | 0.7878 | NA | NA | NA |
| CCEIT vs Isokinetic | 1 | 0.15 | 0.3049 | -0.2301 | 0.4005 | -0.6306 | -1.36 | 0.1728 |
| CCEIT vs Isometric | 2 | 0.26 | 0.2008 | -0.2924 | 0.3785 | -0.6709 | -1.91 | 0.0565 |
| CIT vs CON | 4 | 0.89 | -0.8608 | -0.8716 | -0.7697 | -0.1019 | -0.14 | 0.8871 |
| CIT vs EIT | 3 | 0.92 | 0.0731 | 0.084 | -0.047 | 0.131 | 0.15 | 0.882 |
| CIT vs High speed | 0 | 0 | 0.4888 | NA | 0.4888 | NA | NA | NA |
| CIT vs Isokinetic | 1 | 0.18 | 0.0059 | -0.4501 | 0.1064 | -0.5565 | -0.84 | 0.4029 |
| CIT vs Isometric | 0 | 0 | -0.0982 | NA | -0.0982 | NA | NA | NA |
| EIT vs CON | 2 | 0.75 | -0.9339 | -0.9038 | -1.0237 | 0.1199 | 0.19 | 0.8471 |
| High speed vs CON | 3 | 0.77 | -1.3496 | -1.842 | 0.2767 | -2.1187 | -3.26 | 0.0011 |
| Isokinetic vs CON | 8 | 0.72 | -0.8667 | -1.0408 | -0.4187 | -0.622 | -1.92 | 0.055 |
| Isometric vs CON | 9 | 0.83 | -0.7626 | -0.8866 | -0.1738 | -0.7128 | -2 | 0.0453 |
| EIT vs High speed | 0 | 0 | 0.4157 | NA | 0.4157 | NA | NA | NA |
| EIT vs Isokinetic | 0 | 0 | -0.0672 | NA | -0.0672 | NA | NA | NA |
| EIT vs Isometric | 0 | 0 | -0.1713 | NA | -0.1713 | NA | NA | NA |
| High speed vs Isokinetic | 0 | 0 | -0.4829 | NA | -0.4829 | NA | NA | NA |
| High speed vs Isometric | 2 | 0.69 | -0.587 | -0.2394 | -1.3767 | 1.1373 | 1.9 | 0.0574 |
| Isokinetic vs Isometric | 3 | 0.48 | -0.1041 | -0.2735 | 0.0545 | -0.328 | -0.93 | 0.3507 |

NA not available, k Number of studies providing direct evidence, prop Direct evidence proportion, nma Estimated treatment effect in network meta-analysis, direct Estimated treatment effect derived from direct evidence, indir. Estimated treatment effect derived from indirect evidence, Diff difference (direct versus indirect), z z-value of test for disagreement (direct versus indirect), p p-value of test for disagreement (direct versus indirect), ACCEIT Combined Concentric-Eccentric Isotonic Training in water; CCEIT Combined Concentric-Eccentric Isotonic Training; CIT Concentric Isotonic Training; CON Control group; EIT Eccentric Isotonic Training; High speed Combined Concentric-Eccentric Isotonic Training, as fast as possible; Isokinetic Combined Concentric-Eccentric Isokinetic Training; Isometric Isometric muscle contraction.

**Table 6.3.2 Details of SIDE splitting results (Stiffness)**

| **comparison** | **k** | **prop** | **nma** | **direct** | **indir.** | **Diff** | **z** | **p-value** |
| --- | --- | --- | --- | --- | --- | --- | --- | --- |
| ACCEIT vs CCEIT | 1 | 0.47 | -0.1082 | -0.3842 | 0.1365 | -0.5207 | -1.32 | 0.1852 |
| ACCEIT vs CIT | 0 | 0 | -0.2205 | NA | -0.2205 | NA | NA | NA |
| ACCEIT vs CON | 2 | 0.89 | -0.4576 | -0.3612 | -1.2152 | 0.854 | 1.47 | 0.1408 |
| ACCEIT vs EIT | 0 | 0 | -0.3387 | NA | -0.3387 | NA | NA | NA |
| ACCEIT vs High speed | 0 | 0 | 0.8008 | NA | 0.8008 | NA | NA | NA |
| ACCEIT vs Isokinetic | 0 | 0 | 0.0102 | NA | 0.0102 | NA | NA | NA |
| ACCEIT vs Isometric | 0 | 0 | 0.0895 | NA | 0.0895 | NA | NA | NA |
| CCEIT vs CIT | 0 | 0 | -0.1122 | NA | -0.1122 | NA | NA | NA |
| CCEIT vs CON | 13 | 0.95 | -0.3494 | -0.3559 | -0.2171 | -0.1388 | -0.29 | 0.7725 |
| CCEIT vs EIT | 0 | 0 | -0.2305 | NA | -0.2305 | NA | NA | NA |
| CCEIT vs High speed | 0 | 0 | 0.9091 | NA | 0.9091 | NA | NA | NA |
| CCEIT vs Isokinetic | 0 | 0 | 0.1185 | NA | 0.1185 | NA | NA | NA |
| CCEIT vs Isometric | 1 | 0.32 | 0.1977 | 0.2305 | 0.1826 | 0.0479 | 0.14 | 0.8913 |
| CIT vs CON | 2 | 0.94 | -0.2371 | -0.3524 | 1.6243 | -1.9767 | -2.45 | 0.0145 |
| CIT vs EIT | 2 | 0.91 | -0.1183 | -0.0044 | -1.3023 | 1.2979 | 1.74 | 0.0824 |
| CIT vs High speed | 0 | 0 | 1.0213 | NA | 1.0213 | NA | NA | NA |
| CIT vs Isokinetic | 0 | 0 | 0.2307 | NA | 0.2307 | NA | NA | NA |
| CIT vs Isometric | 0 | 0 | 0.31 | NA | 0.31 | NA | NA | NA |
| EIT vs CON | 1 | 0.65 | -0.1189 | 0.343 | -0.9745 | 1.3175 | 2.61 | 0.009 |
| High speed vs CON | 2 | 0.82 | -1.2584 | -1.3319 | -0.9215 | -0.4104 | -0.56 | 0.5785 |
| Isokinetic vs CON | 5 | 0.84 | -0.4678 | -0.4529 | -0.5436 | 0.0907 | 0.25 | 0.8061 |
| Isometric vs CON | 4 | 0.75 | -0.5471 | -0.5377 | -0.5756 | 0.0379 | 0.12 | 0.9063 |
| EIT vs High speed | 0 | 0 | 1.1396 | NA | 1.1396 | NA | NA | NA |
| EIT vs Isokinetic | 0 | 0 | 0.349 | NA | 0.349 | NA | NA | NA |
| EIT vs Isometric | 0 | 0 | 0.4282 | NA | 0.4282 | NA | NA | NA |
| High speed vs Isokinetic | 0 | 0 | -0.7906 | NA | -0.7906 | NA | NA | NA |
| High speed vs Isometric | 1 | 0.67 | -0.7113 | -0.5741 | -0.9855 | 0.4114 | 0.67 | 0.5009 |
| Isokinetic vs Isometric | 1 | 0.34 | 0.0793 | 0.019 | 0.1098 | -0.0907 | -0.25 | 0.8061 |

NA not available, k Number of studies providing direct evidence, prop Direct evidence proportion, nma Estimated treatment effect in network meta-analysis, direct Estimated treatment effect derived from direct evidence, indir. Estimated treatment effect derived from indirect evidence, Dif difference (direct versus indirect), z z-value of test for disagreement (direct versus indirect), p p-value of test for disagreement (direct versus indirect), ACCEIT Combined Concentric-Eccentric Isotonic Training in water; CCEIT Combined Concentric-Eccentric Isotonic Training; CIT Concentric Isotonic Training; CON Control group; EIT Eccentric Isotonic Training; High speed Combined Concentric-Eccentric Isotonic Training, as fast as possible; Isokinetic Combined Concentric-Eccentric Isokinetic Training; Isometric Isometric muscle contraction.

**Table 6.3.3 Details of SIDE splitting results (Function)**

| **Comparison** | **k** | **prop** | **nma** | **direct** | **indir.** | **Diff** | **z** | **p-value** |
| --- | --- | --- | --- | --- | --- | --- | --- | --- |
| ACCEIT vs CCEIT | 1 | 0.26 | -0.3032 | 0 | -0.4075 | 0.4075 | 0.74 | 0.457 |
| ACCEIT vs CIT | 0 | 0 | -0.6201 | NA | -0.6201 | NA | NA | NA |
| ACCEIT vs CON | 4 | 0.93 | 0.2694 | 0.2043 | 1.1299 | -0.9257 | -1.06 | 0.2903 |
| ACCEIT vs EIT | 0 | 0 | -0.4229 | NA | -0.4229 | NA | NA | NA |
| ACCEIT vs High speed | 0 | 0 | -1.4977 | NA | -1.4977 | NA | NA | NA |
| ACCEIT vs Isokinetic | 0 | 0 | -1.0337 | NA | -1.0337 | NA | NA | NA |
| ACCEIT vs Isometric | 0 | 0 | -0.6919 | NA | -0.6919 | NA | NA | NA |
| CCEIT vs CIT | 0 | 0 | -0.317 | NA | -0.317 | NA | NA | NA |
| CCEIT vs CON | 24 | 0.95 | 0.5726 | 0.537 | 1.234 | -0.697 | -1.53 | 0.1264 |
| CCEIT vs EIT | 0 | 0 | -0.1197 | NA | -0.1197 | NA | NA | NA |
| CCEIT vs High speed | 0 | 0 | -1.1945 | NA | -1.1945 | NA | NA | NA |
| CCEIT vs Isokinetic | 0 | 0 | -0.7305 | NA | -0.7305 | NA | NA | NA |
| CCEIT vs Isometric | 2 | 0.34 | -0.3887 | -0.0272 | -0.5735 | 0.5463 | 1.31 | 0.19 |
| CIT vs CON | 2 | 0.87 | 0.8895 | 0.892 | 0.8729 | 0.019 | 0.02 | 0.986 |
| CIT vs EIT | 1 | 1 | 0.1972 | 0.1972 | NA | NA | NA | NA |
| CIT vs High speed | 0 | 0 | -0.8775 | NA | -0.8775 | NA | NA | NA |
| CIT vs Isokinetic | 1 | 0.43 | -0.4135 | -0.0775 | -0.6626 | 0.5851 | 0.7 | 0.4846 |
| CIT vs Isometric | 0 | 0 | -0.0718 | NA | -0.0718 | NA | NA | NA |
| EIT vs CON | 0 | 0 | 0.6923 | NA | 0.6923 | NA | NA | NA |
| High speed vs CON | 2 | 0.8 | 1.767 | 2.1462 | 0.2934 | 1.8528 | 1.91 | 0.0562 |
| Isokinetic vs CON | 4 | 0.85 | 1.303 | 1.3779 | 0.8652 | 0.5127 | 0.64 | 0.5191 |
| Isometric vs CON | 6 | 0.78 | 0.9613 | 1.1158 | 0.4272 | 0.6886 | 1.57 | 0.1173 |
| EIT vs High speed | 0 | 0 | -1.0748 | NA | -1.0748 | NA | NA | NA |
| EIT vs Isokinetic | 0 | 0 | -0.6108 | NA | -0.6108 | NA | NA | NA |
| EIT vs Isometric | 0 | 0 | -0.269 | NA | -0.269 | NA | NA | NA |
| High speed vs Isokinetic | 0 | 0 | 0.464 | NA | 0.464 | NA | NA | NA |
| High speed vs Isometric | 1 | 0.6 | 0.8057 | 0.6319 | 1.0613 | -0.4294 | -0.53 | 0.5972 |
| Isokinetic vs Isometric | 1 | 0.39 | 0.3417 | 0.4325 | 0.2837 | 0.1488 | 0.23 | 0.8171 |

NA not available, k Number of studies providing direct evidence, prop Direct evidence proportion, nma Estimated treatment effect in network meta-analysis, direct Estimated treatment effect derived from direct evidence, indir. Estimated treatment effect derived from indirect evidence, Dif difference (direct versus indirect), z z-value of test for disagreement (direct versus indirect), p p-value of test for disagreement (direct versus indirect), ACCEIT Combined Concentric-Eccentric Isotonic Training in water; CCEIT Combined Concentric-Eccentric Isotonic Training; CIT Concentric Isotonic Training; CON Control group; EIT Eccentric Isotonic Training; High speed Combined Concentric-Eccentric Isotonic Training, as fast as possible; Isokinetic Combined Concentric-Eccentric Isokinetic Training; Isometric Isometric muscle contraction.

# Supplementary 7: Network plot

Figure 7.1: Network plot of pain. The size of the nodes corresponds to the number of participants randomized to each resistance training type. Resistance training type with direct comparisons are linked with a line; its thickness corresponds to the number of trials evaluating the comparison. ACCEIT Combined Concentric-Eccentric Isotonic Training in water; CCEIT Combined Concentric-Eccentric Isotonic Training; CIT Concentric Isotonic Training; CON Control group; EIT Eccentric Isotonic Training; High speed Combined Concentric-Eccentric Isotonic Training, as fast as possible; Isokinetic Combined Concentric-Eccentric Isokinetic Training; Isometric Isometric muscle contraction.

Figure 7.2: Network plot of stiffness. The size of the nodes corresponds to the number of participants randomized to each resistance training type. Resistance training type with direct comparisons are linked with a line; its thickness corresponds to the number of trials evaluating the comparison. ACCEIT Combined Concentric-Eccentric Isotonic Training in water; CCEIT Combined Concentric-Eccentric Isotonic Training; CIT Concentric Isotonic Training; CON Control group; EIT Eccentric Isotonic Training; High speed Combined Concentric-Eccentric Isotonic Training, as fast as possible; Isokinetic Combined Concentric-Eccentric Isokinetic Training; Isometric Isometric muscle contraction.

Figure 7.3: Network plot of function. The size of the nodes corresponds to the number of participants randomized to each resistance training type. Resistance training type with direct comparisons are linked with a line; its thickness corresponds to the number of trials evaluating the comparison. ACCEIT Combined Concentric-Eccentric Isotonic Training in water; CCEIT Combined Concentric-Eccentric Isotonic Training; CIT Concentric Isotonic Training; CON Control group; EIT Eccentric Isotonic Training; High speed Combined Concentric-Eccentric Isotonic Training, as fast as possible; Isokinetic Combined Concentric-Eccentric Isokinetic Training; Isometric Isometric muscle contraction.

# Supplementary 8: League table

Table 8.1 The league table of pain

| **High speed**  **(0.96)** | NA | NA | NA | -0.24 (-0.89; 0.41) | NA | NA | **-1.84 (-2.46; -1.23)** |
| --- | --- | --- | --- | --- | --- | --- | --- |
| -0.42 (-1.17; 0.34) | **EIT**  **(0.69)** | NA | -0.08 (-0.58; 0.41) | NA | NA | NA | **-0.90 (-1.51; -0.29)** |
| -0.48 (-1.08; 0.11) | -0.07 (-0.66; 0.52) | **Isokinetic**  **(0.66)** | 0.45 (-0.73; 1.63) | -0.27 (-0.77; 0.22) | 0.19 (-0.65; 1.03) | 0.23 (-0.60; 1.07) | **-1.04 (-1.38; -0.70)** |
| -0.49 (-1.18; 0.20) | -0.07 (-0.55; 0.40) | -0.01 (-0.51; 0.50) | **CIT**  **(0.62)** | NA | NA | NA | **-0.87 (-1.33; -0.41)** |
| **-0.59 (-1.13; -0.05)** | -0.17 (-0.76; 0.42) | -0.10 (-0.45; 0.24) | -0.10 (-0.60; 0.41) | **Isometric**  **(0.52)** | NA | 0.29 (-0.30; 0.88) | **-0.89 (-1.18; -0.60)** |
| **-0.79 (-1.43; -0.15)** | -0.37 (-1.01; 0.26) | -0.31 (-0.73; 0.12) | -0.30 (-0.86; 0.26) | -0.20 (-0.64; 0.24) | **ACCEIT**  **(0.29)** | -0.45 (-1.28; 0.38) | -0.40 (-0.81; 0.01) |
| **-0.79 (-1.35; -0.23)** | -0.37 (-0.93; 0.18) | -0.30 (-0.63; 0.02) | -0.30 (-0.77; 0.17) | -0.20 (-0.51; 0.10) | 0.00 (-0.39; 0.39) | **CCEIT**  **(0.26)** | **-0.55 (-0.73; -0.38)** |
| **-1.35 (-1.89; -0.81)** | **-0.93 (-1.46; -0.41)** | **-0.87 (-1.15; -0.58)** | **-0.86 (-1.29; -0.43)** | **-0.76 (-1.03; -0.50)** | **-0.56 (-0.92; -0.20)** | **-0.56 (-0.74; -0.39)** | **CON**  **(0.00)** |

All results are presented in the form of SMD (95% CrI). Treatment types are ranked according to the SUCRA for pain with the best from left to right. The results of the network meta-analysis are showed in the lower left part, and results from pairwise comparisons in the upper right half (if available). Cells shown in bold indicate significant results. NA not available, SMD standardized mean difference, CrI Credible Interval, ACCEIT Combined Concentric-Eccentric Isotonic Training in water; CCEIT Combined Concentric-Eccentric Isotonic Training; CIT Concentric Isotonic Training; CON Control group; EIT Eccentric Isotonic Training; High speed Combined Concentric-Eccentric Isotonic Training, as fast as possible; Isokinetic Combined Concentric-Eccentric Isokinetic Training; Isometric Isometric muscle contraction.

Table 8.2 The league table of stiffness

| **High speed**  **(1.00)** | -0.57 (-1.27; 0.12) | NA | NA | NA | NA | NA | **-1.33 (-1.94; -0.72)** |
| --- | --- | --- | --- | --- | --- | --- | --- |
| **-0.71 (-1.28; -0.15)** | **Isometric**  **(0.72)** | -0.02 (-0.61; 0.57) | NA | -0.23 (-0.80; 0.34) | NA | NA | **-0.54 (-0.85; -0.22)** |
| **-0.79 (-1.40; -0.18)** | -0.08 (-0.42; 0.26) | **Isokinetic**  **(0.62)** | NA | NA | NA | NA | **-0.45 (-0.75; -0.16)** |
| **-0.80 (-1.46; -0.14)** | -0.09 (-0.54; 0.36) | -0.01 (-0.46; 0.44) | **ACCEIT**  **(0.60)** | -0.38 (-0.95; 0.18) | NA | NA | -0.36 (-0.74; 0.02) |
| **-0.91 (-1.49; -0.32)** | -0.20 (-0.52; 0.12) | -0.12 (-0.45; 0.21) | -0.11 (-0.49; 0.28) | **CCEIT**  **(0.45)** | NA | NA | **-0.36 (-0.56; -0.15)** |
| **-1.02 (-1.69; -0.35)** | -0.31 (-0.77; 0.15) | -0.23 (-0.69; 0.23) | -0.22 (-0.74; 0.30) | -0.11 (-0.53; 0.31) | **CIT**  **(0.34)** | -0.00 (-0.44; 0.43) | -0.35 (-0.73; 0.03) |
| **-1.14 (-1.87; -0.41)** | -0.43 (-0.97; 0.12) | -0.35 (-0.89; 0.19) | -0.34 (-0.93; 0.25) | -0.23 (-0.74; 0.28) | -0.12 (-0.53; 0.30) | **EIT**  **(0.21)** | 0.34 (-0.24; 0.93) |
| **-1.26 (-1.81; -0.70)** | **-0.55 (-0.82; -0.27)** | **-0.47 (-0.74; -0.20)** | **-0.46 (-0.82; -0.10)** | **-0.35 (-0.55; -0.15)** | -0.24 (-0.61; 0.13) | -0.12 (-0.59; 0.35) | **CON**  **(0.06)** |

All results are presented in the form of SMD (95% CrI). Treatment types are ranked according to the SUCRA for stiffness with the best from left to right. The results of the network meta-analysis are showed in the lower left part, and results from pairwise comparisons in the upper right half (if available). Cells shown in bold indicate significant results. NA not available, SMD standardized mean difference, CrI Credible Interval, ACCEIT Combined Concentric-Eccentric Isotonic Training in water; CCEIT Combined Concentric-Eccentric Isotonic Training; CIT Concentric Isotonic Training; CON Control group; EIT Eccentric Isotonic Training; High speed Combined Concentric-Eccentric Isotonic Training, as fast as possible; Isokinetic Combined Concentric-Eccentric Isokinetic Training; Isometric Isometric muscle contraction.

Table 8.3 The league table of function

| **High speed**  **(0.96)** | NA | 0.63 (-0.38; 1.65) | NA | NA | NA | NA | **2.15 ( 1.29; 3.01)** |
| --- | --- | --- | --- | --- | --- | --- | --- |
| 0.46 (-0.47; 1.40) | **Isokinetic**  **(0.81)** | 0.43 (-0.55; 1.42) | 0.08 (-1.17; 1.32) | NA | NA | NA | **1.38 ( 0.78; 1.97)** |
| 0.81 ( 0.02; 1.59) | 0.34 (-0.27; 0.96) | **Isometric**  **(0.62)** | NA | NA | 0.03 (-0.64; 0.69) | NA | **1.12 ( 0.71; 1.52)** |
| 0.88 (-0.17; 1.92) | 0.41 (-0.40; 1.22) | 0.07 (-0.72; 0.86) | **CIT**  **(0.57)** | 0.20 (-0.77; 1.16) | NA | NA | **0.89 ( 0.13; 1.65)** |
| 1.07 (-0.35; 2.50) | 0.61 (-0.65; 1.87) | 0.27 (-0.98; 1.51) | 0.20 (-0.77; 1.16) | **EIT**  **(0.44)** | NA | NA | NA |
| **1.19 ( 0.41; 1.98)** | **0.73 ( 0.15; 1.31)** | **0.39 ( 0.00; 0.78)** | 0.32 (-0.42; 1.05) | 0.12 (-1.09; 1.33) | **CCEIT**  **(0.36)** | -0.00 (-0.93; 0.93) | **0.54 ( 0.34; 0.74)** |
| **1.50 ( 0.61; 2.38)** | **1.03 ( 0.33; 1.74)** | **0.69 ( 0.13; 1.26)** | 0.62 (-0.21; 1.45) | 0.42 (-0.85; 1.70) | 0.30 (-0.17; 0.77) | **ACCEIT**  **(0.19)** | 0.20 (-0.25; 0.66) |
| **1.77 ( 1.00; 2.53)** | **1.30 ( 0.75; 1.85)** | **0.96 ( 0.60; 1.32)** | **0.89 ( 0.18; 1.60)** | 0.69 (-0.51; 1.89) | **0.57 ( 0.38; 0.77)** | 0.27 (-0.17; 0.71) | **CON**  **(0.04)** |

All results are presented in the form of SMD (95% CrI). Treatment types are ranked according to the SUCRA for function with the best from left to right. The results of the network meta-analysis are showed in the lower left part, and results from pairwise comparisons in the upper right half (if available). Cells shown in bold indicate significant results. NA not available, SMD standardized mean difference, CrI Credible Interval, ACCEIT Combined Concentric-Eccentric Isotonic Training in water; CCEIT Combined Concentric-Eccentric Isotonic Training; CIT Concentric Isotonic Training; CON Control group; EIT Eccentric Isotonic Training; High speed Combined Concentric-Eccentric Isotonic Training, as fast as possible; Isokinetic Combined Concentric-Eccentric Isokinetic Training; Isometric Isometric muscle contraction.

# Supplementary 9: Publication bias

Figure 9.1 The funnel plot of pain. The result of Egger test showed the p=0.268. Therefore, no small study effect was found for the pain.

Figure 9.2 The funnel plot of stiffness. The result of Egger test showed the p=0.799. Therefore, no small study effect was found for the stiffness.

Figure 9.3 The funnel plot of function. The result of Egger test showed the p=0.212. Therefore, no small study effect was found for the function.

Supplementary 10: Grading the evidence of the network meta-analysis using CINeMA

## 10.1 CINeMA for Pain

| **Comparison** | **Number of studies** | **Within-study bias** | **Reporting bias** | **Indirectness** | **Imprecision** | **Heterogeneity** | **Incoherence** | **Confidence rating** | **Reason(s) for downgrading** |
| --- | --- | --- | --- | --- | --- | --- | --- | --- | --- |
| ACCEIT:CCEIT | 2 | No concerns | Low risk | No concerns | Major concerns | No concerns | No concerns | Low | ["Imprecision"] |
| ACCEIT:CON | 4 | No concerns | Low risk | No concerns | No concerns | Major concerns | No concerns | Low | ["Heterogeneity"] |
| CCEIT:CON | 25 | No concerns | Low risk | No concerns | No concerns | Major concerns | No concerns | Low | ["Heterogeneity"] |
| CCEIT:Isokinetic | 1 | Some concerns | Low risk | No concerns | No concerns | Major concerns | No concerns | Very low | ["Within-study bias","Heterogeneity"] |
| CCEIT:Isometric | 2 | Some concerns | Low risk | No concerns | Major concerns | No concerns | Major concerns | Very low | ["Within-study bias","Imprecision","Incoherence"] |
| CIT:CON | 4 | Some concerns | Low risk | No concerns | No concerns | No concerns | No concerns | Moderate | ["Within-study bias"] |
| CIT:EIT | 3 | Some concerns | Low risk | No concerns | Major concerns | No concerns | No concerns | Very low | ["Within-study bias","Imprecision"] |
| CIT:Isokinetic | 1 | Some concerns | Low risk | No concerns | Major concerns | No concerns | No concerns | Low | ["Within-study bias","Imprecision"] |
| CON:EIT | 2 | Some concerns | Low risk | No concerns | No concerns | No concerns | No concerns | Moderate | ["Within-study bias"] |
| CON:High speed | 3 | Some concerns | Low risk | No concerns | No concerns | No concerns | Major concerns | Very low | ["Within-study bias","Incoherence"] |
| CON:Isokinetic | 8 | Some concerns | Low risk | No concerns | No concerns | No concerns | No concerns | Moderate | ["Within-study bias"] |
| CON:Isometric | 9 | Some concerns | Low risk | No concerns | No concerns | No concerns | Major concerns | Very low | ["Within-study bias","Incoherence"] |
| High speed:Isometric | 2 | Some concerns | Low risk | No concerns | No concerns | Major concerns | Major concerns | Very low | ["Within-study bias","Heterogeneity","Incoherence"] |
| Isokinetic:Isometric | 3 | Some concerns | Low risk | No concerns | Major concerns | No concerns | No concerns | Very low | ["Within-study bias","Imprecision"] |
| ACCEIT:CIT | 0 | Some concerns | Low risk | No concerns | Major concerns | No concerns | Major concerns | Very low | ["Within-study bias","Imprecision","Incoherence"] |
| ACCEIT:EIT | 0 | Some concerns | Low risk | No concerns | Major concerns | No concerns | Major concerns | Very low | ["Within-study bias","Imprecision","Incoherence"] |
| ACCEIT:High speed | 0 | Some concerns | Low risk | No concerns | No concerns | Major concerns | Major concerns | Very low | ["Within-study bias","Heterogeneity","Incoherence"] |
| ACCEIT:Isokinetic | 0 | Some concerns | Low risk | No concerns | Major concerns | No concerns | Major concerns | Very low | ["Within-study bias","Imprecision","Incoherence"] |
| ACCEIT:Isometric | 0 | Some concerns | Low risk | No concerns | Major concerns | No concerns | Major concerns | Very low | ["Within-study bias","Imprecision","Incoherence"] |
| CCEIT:CIT | 0 | Some concerns | Low risk | No concerns | Major concerns | No concerns | Major concerns | Very low | ["Within-study bias","Imprecision","Incoherence"] |
| CCEIT:EIT | 0 | Some concerns | Low risk | No concerns | Major concerns | No concerns | Major concerns | Very low | ["Within-study bias","Imprecision","Incoherence"] |
| CCEIT:High speed | 0 | Some concerns | Low risk | No concerns | No concerns | Major concerns | Major concerns | Very low | ["Within-study bias","Heterogeneity","Incoherence"] |
| CIT:High speed | 0 | Some concerns | Low risk | No concerns | Major concerns | No concerns | Major concerns | Very low | ["Within-study bias","Imprecision","Incoherence"] |
| CIT:Isometric | 0 | Some concerns | Low risk | No concerns | Major concerns | No concerns | Major concerns | Very low | ["Within-study bias","Imprecision","Incoherence"] |
| EIT:High speed | 0 | Some concerns | Low risk | No concerns | Major concerns | No concerns | Major concerns | Very low | ["Within-study bias","Imprecision","Incoherence"] |
| EIT:Isokinetic | 0 | Some concerns | Low risk | No concerns | Major concerns | No concerns | Major concerns | Very low | ["Within-study bias","Imprecision","Incoherence"] |
| EIT:Isometric | 0 | Some concerns | Low risk | No concerns | Major concerns | No concerns | Major concerns | Very low | ["Within-study bias","Imprecision","Incoherence"] |
| High speed:Isokinetic | 0 | Some concerns | Low risk | No concerns | Major concerns | No concerns | Major concerns | Very low | ["Within-study bias","Imprecision","Incoherence"] |

## 10.2 CINeMA for Stiffness

| **Comparison** | **Number of studies** | **Within-study bias** | **Reporting bias** | **Indirectness** | **Imprecision** | **Heterogeneity** | **Incoherence** | **Confidence rating** | **Reason(s) for downgrading** |
| --- | --- | --- | --- | --- | --- | --- | --- | --- | --- |
| ACCEIT:CCEIT | 1 | Some concerns | Low risk | No concerns | Major concerns | No concerns | No concerns | Very low | ["Within-study bias","Imprecision"] |
| ACCEIT:CON | 2 | Some concerns | Low risk | No concerns | No concerns | Major concerns | No concerns | Very low | ["Within-study bias","Heterogeneity"] |
| CCEIT:CON | 13 | Some concerns | Low risk | No concerns | No concerns | Major concerns | No concerns | Very low | ["Within-study bias","Heterogeneity"] |
| CCEIT:Isometric | 1 | Some concerns | Low risk | No concerns | Major concerns | No concerns | No concerns | Very low | ["Within-study bias","Imprecision"] |
| CIT:CON | 2 | Some concerns | Low risk | No concerns | Major concerns | No concerns | Major concerns | Very low | ["Within-study bias","Imprecision","Incoherence"] |
| CIT:EIT | 2 | Some concerns | Low risk | No concerns | Major concerns | No concerns | No concerns | Very low | ["Within-study bias","Imprecision"] |
| CON:EIT | 1 | Some concerns | Low risk | No concerns | Major concerns | No concerns | Major concerns | Very low | ["Within-study bias","Imprecision","Incoherence"] |
| CON:High speed | 2 | Some concerns | Low risk | No concerns | No concerns | No concerns | No concerns | Moderate | ["Within-study bias"] |
| CON:Isokinetic | 5 | Some concerns | Low risk | No concerns | No concerns | No concerns | No concerns | Moderate | ["Within-study bias"] |
| CON:Isometric | 4 | Some concerns | Low risk | No concerns | No concerns | No concerns | No concerns | Moderate | ["Within-study bias"] |
| High speed:Isometric | 1 | Some concerns | Low risk | No concerns | No concerns | No concerns | No concerns | Moderate | ["Within-study bias"] |
| Isokinetic:Isometric | 1 | Some concerns | Low risk | No concerns | Major concerns | No concerns | No concerns | Very low | ["Within-study bias","Imprecision"] |
| ACCEIT:CIT | 0 | Some concerns | Low risk | No concerns | Major concerns | No concerns | No concerns | Very low | ["Within-study bias","Imprecision"] |
| ACCEIT:EIT | 0 | Some concerns | Low risk | No concerns | Major concerns | No concerns | No concerns | Very low | ["Within-study bias","Imprecision"] |
| ACCEIT:High speed | 0 | Some concerns | Low risk | No concerns | No concerns | No concerns | No concerns | Moderate | ["Within-study bias"] |
| ACCEIT:Isokinetic | 0 | Some concerns | Low risk | No concerns | Major concerns | No concerns | No concerns | Very low | ["Within-study bias","Imprecision"] |
| ACCEIT:Isometric | 0 | Some concerns | Low risk | No concerns | Major concerns | No concerns | No concerns | Very low | ["Within-study bias","Imprecision"] |
| CCEIT:CIT | 0 | Some concerns | Low risk | No concerns | Major concerns | No concerns | No concerns | Very low | ["Within-study bias","Imprecision"] |
| CCEIT:EIT | 0 | Some concerns | Low risk | No concerns | Major concerns | No concerns | No concerns | Very low | ["Within-study bias","Imprecision"] |
| CCEIT:High speed | 0 | Some concerns | Low risk | No concerns | No concerns | No concerns | No concerns | Moderate | ["Within-study bias"] |
| CCEIT:Isokinetic | 0 | Some concerns | Low risk | No concerns | Major concerns | No concerns | No concerns | Very low | ["Within-study bias","Imprecision"] |
| CIT:High speed | 0 | Some concerns | Low risk | No concerns | No concerns | No concerns | No concerns | Moderate | ["Within-study bias"] |
| CIT:Isokinetic | 0 | Some concerns | Low risk | No concerns | Major concerns | No concerns | No concerns | Very low | ["Within-study bias","Imprecision"] |
| CIT:Isometric | 0 | Some concerns | Low risk | No concerns | Major concerns | No concerns | No concerns | Very low | ["Within-study bias","Imprecision"] |
| EIT:High speed | 0 | Some concerns | Low risk | No concerns | No concerns | No concerns | No concerns | Moderate | ["Within-study bias"] |
| EIT:Isokinetic | 0 | Some concerns | Low risk | No concerns | Major concerns | No concerns | No concerns | Very low | ["Within-study bias","Imprecision"] |
| EIT:Isometric | 0 | Some concerns | Low risk | No concerns | Major concerns | No concerns | No concerns | Very low | ["Within-study bias","Imprecision"] |
| High speed:Isokinetic | 0 | Some concerns | Low risk | No concerns | No concerns | No concerns | No concerns | Moderate | ["Within-study bias"] |

## 10.3 CINeMA for Function

| **Comparison** | **Number of studies** | **Within-study bias** | **Reporting bias** | **Indirectness** | **Imprecision** | **Heterogeneity** | **Incoherence** | **Confidence rating** | **Reason(s) for downgrading** |
| --- | --- | --- | --- | --- | --- | --- | --- | --- | --- |
| ACCEIT:CCEIT | 1 | Some concerns | Low risk | No concerns | Major concerns | No concerns | No concerns | Very low | ["Within-study bias","Imprecision"] |
| ACCEIT:CON | 4 | Some concerns | Low risk | No concerns | Major concerns | No concerns | No concerns | Very low | ["Within-study bias","Imprecision"] |
| CCEIT:CON | 24 | No concerns | Low risk | No concerns | No concerns | Major concerns | No concerns | Low | ["Heterogeneity"] |
| CCEIT:Isometric | 2 | Some concerns | Low risk | No concerns | Major concerns | No concerns | No concerns | Very low | ["Within-study bias","Imprecision"] |
| CIT:CON | 2 | Some concerns | Low risk | No concerns | No concerns | Major concerns | No concerns | Very low | ["Within-study bias","Heterogeneity"] |
| CIT:EIT | 1 | No concerns | Low risk | No concerns | Major concerns | No concerns | No concerns | Low | ["Imprecision"] |
| CIT:Isokinetic | 1 | Some concerns | Low risk | No concerns | Major concerns | No concerns | No concerns | Very low | ["Within-study bias","Imprecision"] |
| CON:High speed | 2 | Some concerns | Low risk | No concerns | No concerns | No concerns | Major concerns | Very low | ["Within-study bias","Incoherence"] |
| CON:Isokinetic | 4 | Some concerns | Low risk | No concerns | No concerns | No concerns | No concerns | Moderate | ["Within-study bias"] |
| CON:Isometric | 5 | Some concerns | Low risk | No concerns | No concerns | Major concerns | No concerns | Very low | ["Within-study bias","Heterogeneity"] |
| High speed:Isometric | 1 | Some concerns | Low risk | No concerns | No concerns | Major concerns | No concerns | Very low | ["Within-study bias","Heterogeneity"] |
| Isokinetic:Isometric | 1 | Some concerns | Low risk | No concerns | Major concerns | No concerns | No concerns | Very low | ["Within-study bias","Imprecision"] |
| ACCEIT:CIT | 0 | Some concerns | Low risk | No concerns | Major concerns | No concerns | No concerns | Very low | ["Within-study bias","Imprecision"] |
| ACCEIT:EIT | 0 | No concerns | Low risk | No concerns | Major concerns | No concerns | No concerns | Low | ["Imprecision"] |
| ACCEIT:High speed | 0 | Some concerns | Low risk | No concerns | No concerns | No concerns | No concerns | Moderate | ["Within-study bias"] |
| ACCEIT:Isokinetic | 0 | Some concerns | Low risk | No concerns | No concerns | Major concerns | No concerns | Very low | ["Within-study bias","Heterogeneity"] |
| ACCEIT:Isometric | 0 | Some concerns | Low risk | No concerns | Major concerns | No concerns | No concerns | Very low | ["Within-study bias","Imprecision"] |
| CCEIT:CIT | 0 | Some concerns | Low risk | No concerns | Major concerns | No concerns | No concerns | Very low | ["Within-study bias","Imprecision"] |
| CCEIT:EIT | 0 | No concerns | Low risk | No concerns | Major concerns | No concerns | No concerns | Low | ["Imprecision"] |
| CCEIT:High speed | 0 | Some concerns | Low risk | No concerns | No concerns | Major concerns | No concerns | Very low | ["Within-study bias","Heterogeneity"] |
| CCEIT:Isokinetic | 0 | Some concerns | Low risk | No concerns | No concerns | Major concerns | No concerns | Very low | ["Within-study bias","Heterogeneity"] |
| CIT:High speed | 0 | Some concerns | Low risk | No concerns | Major concerns | No concerns | No concerns | Very low | ["Within-study bias","Imprecision"] |
| CIT:Isometric | 0 | Some concerns | Low risk | No concerns | Major concerns | No concerns | No concerns | Very low | ["Within-study bias","Imprecision"] |
| CON:EIT | 0 | No concerns | Low risk | No concerns | Major concerns | No concerns | No concerns | Low | ["Imprecision"] |
| EIT:High speed | 0 | Some concerns | Low risk | No concerns | Major concerns | No concerns | No concerns | Very low | ["Within-study bias","Imprecision"] |
| EIT:Isokinetic | 0 | No concerns | Low risk | No concerns | Major concerns | No concerns | No concerns | Low | ["Imprecision"] |
| EIT:Isometric | 0 | Some concerns | Low risk | No concerns | Major concerns | No concerns | No concerns | Very low | ["Within-study bias","Imprecision"] |
| High speed:Isokinetic | 0 | Some concerns | Low risk | No concerns | Major concerns | No concerns | No concerns | Very low | ["Within-study bias","Imprecision"] |

**Supplementary 11:** Assessment of Connectivity, Consistency and Transitivity in Network Meta Dose-Response Analysis

***Connectivity***

Connectivity is a key assumption in network meta-dose analysis, and evidence of unconnectedness may lead to low statistical power and misleading results [3]. Our results show that there is no phenomenon of poor connectivity (Figures 10).

**Figure 11.1**. Treatment-level network (Pain). The first value indicates resistance training and the second one is the corresponding intensity of that intervention (The unit of intensity is percentage of 1RM). RT resistance training.

**Figure 11.2**. Treatment-level network (Pain). The first value indicates resistance training and the second one is the corresponding period of that intervention (The unit of period is week). RT resistance training.

**Figure 11.3**. Treatment-level network (Pain). The first value indicates resistance training and the second one is the corresponding number of repetitions/week of that intervention. RT resistance training.

**Figure 11.4**. Treatment-level network (Stiffness). The first value indicates resistance training and the second one is the corresponding intensity of that intervention. RT resistance training.

**Figure 11.5**. Treatment-level network (Stiffness). The first value indicates resistance training and the second one is the corresponding period of that intervention. RT resistance training.

**Figure 11.6**. Treatment-level network (Stiffness). The first value indicates resistance training and the second one is the corresponding number of repetition/week of that intervention. RT resistance training.

**Figure 11.7**. Treatment-level network (Function). The first value indicates resistance training and the second one is the corresponding intensity of that intervention. RT resistance training.

**Figure 11.8**. Treatment-level network (Function). The first value indicates resistance training and the second one is the corresponding period of that intervention. RT resistance training.

**Figure 11.9**. Treatment-level network (Function). The first value indicates resistance training and the second one is the corresponding number of repetition/week of that intervention. RT resistance training.

**Consistency**

We analyzed the data with the consistency model and the unrelated mean effect model, and compared the differences in the deviation, the number of estimated parameters in the network, and the Deviance Informative Criterion (DIC) indicators of the two models. If these are similar, it means that our research has good consistency [4]. Comparison of these parameters indicated good consistency across models (Table 10.1).

**Table 11.1.** Consistent and UME models fit comparison

|  | **Model** | **pD** | **Residual deviance** | **Deviance** | **DIC** | **SD** |
| --- | --- | --- | --- | --- | --- | --- |
| **Pain** |  |  |  |  |  |  |
| Intensity | Consistent | 89.1 | 108.726 | 272.189 | 360.5 | 0.385 |
|  | UME | 91.1 | 108.948 | 272.411 | 362.8 | 0.488 |
| Period | Consistent | 80.6 | 110.284 | 273.747 | 353.6 | 0.243 |
|  | UME | 85.1 | 109.510 | 270.973 | 355.4 | 0.242 |
| Number of repetitions/week | Consistent | 91.0 | 107.726 | 271.189 | 361.4 | 0.450 |
|  | UME | 96.9 | 107.473 | 270.936 | 367.2 | 0.668 |
| **Stiffness** |  |  |  |  |  |  |
| Intensity | Consistent | 43.0 | 61.051 | 134.710 | 177.3 | 0.154 |
|  | UME | 45.5 | 63.719 | 137.378 | 181.9 | 0.360 |
| Period | Consistent | 47.1 | 63.782 | 137.441 | 184.0 | 0.265 |
|  | UME | 47.5 | 62.997 | 136.656 | 183.5 | 0.409 |
| Number of repetitions/week | Consistent | 43.1 | 61.999 | 135.685 | 177.9 | 0.240 |
|  | UME | 43.5 | 62.365 | 136.023 | 179.0 | 0.299 |
| **Function** |  |  |  |  |  |  |
| Intensity | Consistent | 75.3 | 84.890 | 390.966 | 466.0 | 0.511 |
|  | UME | 75.0 | 84.064 | 390.141 | 464.5 | 0.666 |
| Period | Consistent | 72.2 | 85.44 | 391.520 | 463.2 | 0.431 |
|  | UME | 78.2 | 83.885 | 389.962 | 467.6 | 0.862 |
| Number of repetitions/week | Consistent | 74.3 | 84.835 | 390.912 | 464.8 | 0.560 |
|  | UME | 77.4 | 83.754 | 389.830 | 466.7 | 0.808 |

pD: Number of estimated parameters; DIC: Deviance Informative Criterion; SD: Standard Deviation; UME: Unrelated Mean Effects. Scientific literature indicated that the main indicator to assess the model fit is the DIC. As lower DIC, better fit.

**Transitivity**

We assessed transitivity via MBNMA node-splitting approach. This method splits and compares contributions for a particular treatment contrast into direct and indirect evidence [5]. Similar effects denote good transitivity. Table 10.2 below present the results for transitivity in this meta-analysis.

**Table 11.2.1 Node-splitting analysis of inconsistency (Pain-intensity)**

| **Comparison** | | **p-value** | | **Median** | **2.50%** | **97.50%** |
| --- | --- | --- | --- | --- | --- | --- |
| RT_75 vs RT_70 | | 0.057 | |  |  |  |
| -> direct |  | |  | -0.156 | -0.901 | 0.565 |
| -> indirect |  | |  | -0.015 | -0.04 | -0.001 |
| -> MBNMA | |  | | -0.014 | -0.04 | -0.001 |
|  |  | |  |  |  |  |
| RT_35 vs RT_30 | | 0.059 | |  |  |  |
| -> direct |  | |  | -0.193 | -1.232 | 0.799 |
| -> indirect |  | |  | -0.04 | -0.061 | -0.003 |
| -> MBNMA | |  | | -0.04 | -0.062 | -0.003 |
|  |  | |  |  |  |  |
| RT_85 vs Placebo_0 | | 0.025 | |  |  |  |
| -> direct |  | |  | -0.07 | -0.622 | 0.464 |
| -> indirect |  | |  | -0.9 | -1.121 | -0.707 |
| -> MBNMA | |  | | -0.811 | -1.022 | -0.634 |
|  |  | |  |  |  |  |
| RT_80 vs Placebo_0 | | 0.504 | |  |  |  |
| -> direct |  | |  | -0.733 | -1.294 | -0.157 |
| -> indirect |  | |  | -0.813 | -1.011 | -0.636 |
| -> MBNMA | |  | | -0.797 | -0.991 | -0.627 |
|  |  | |  |  |  |  |
| RT_65 vs Placebo_0 | | 0.299 | |  |  |  |
| -> direct |  | |  | -1.041 | -1.65 | -0.449 |
| -> indirect |  | |  | -0.733 | -0.892 | -0.568 |
| -> MBNMA | |  | | -0.751 | -0.91 | -0.598 |
|  |  | |  |  |  |  |
| RT_60 vs Placebo_0 | | 0.582 | |  |  |  |
| -> direct |  | |  | -0.831 | -1.107 | -0.551 |
| -> indirect |  | |  | -0.71 | -0.892 | -0.531 |
| -> MBNMA | |  | | -0.733 | -0.89 | -0.579 |
|  |  | |  |  |  |  |
| RT_55 vs Placebo_0 | | 0.402 | |  |  |  |
| -> direct |  | |  | -0.525 | -1.017 | -0.045 |
| -> indirect |  | |  | -0.73 | -0.895 | -0.566 |
| -> MBNMA | |  | | -0.712 | -0.875 | -0.556 |
|  |  | |  |  |  |  |
| RT_50 vs Placebo_0 | | 0.06 | |  |  |  |
| -> direct |  | |  | -1.256 | -1.722 | -0.787 |
| -> indirect |  | |  | -0.659 | -0.833 | -0.495 |
| -> MBNMA | |  | | -0.688 | -0.855 | -0.531 |
|  |  | |  |  |  |  |
| RT_40 vs Placebo_0 | | 0.001 | |  |  |  |
| -> direct |  | |  | -2.029 | -2.678 | -1.341 |
| -> indirect |  | |  | -0.545 | -0.719 | -0.409 |
| -> MBNMA | |  | | -0.633 | -0.824 | -0.458 |
|  |  | |  |  |  |  |
| RT_10 vs Placebo_0 | | 0.435 | |  |  |  |
| -> direct |  | |  | -0.488 | -1.562 | 0.559 |
| -> indirect |  | |  | -0.28 | -0.702 | -0.138 |
| -> MBNMA | |  | | -0.292 | -0.707 | -0.141 |

**Table 11.2.2 Node-splitting analysis of inconsistency (Pain-period)**

| Comparison | | p-value | Median | 2.50% | 97.50% |
| --- | --- | --- | --- | --- | --- |
| RT_72 vs Placebo_0 | | 0.034 |  |  |  |
| -> direct |  |  | -0.017 | -0.65 | 0.556 |
| -> indirect |  |  | -0.905 | -1.382 | -0.695 |
| -> MBNMA | |  | -0.804 | -1.049 | -0.634 |
|  |  |  |  |  |  |
| RT_36 vs Placebo_0 | | 0.129 |  |  |  |
| -> direct |  |  | -0.096 | -1.016 | 0.874 |
| -> indirect |  |  | -0.822 | -1.049 | -0.647 |
| -> MBNMA | |  | -0.792 | -0.996 | -0.626 |
|  |  |  |  |  |  |
| RT_24 vs Placebo_0 | | 0.187 |  |  |  |
| -> direct |  |  | -0.338 | -0.937 | 0.23 |
| -> indirect |  |  | -0.821 | -1.024 | -0.643 |
| -> MBNMA | |  | -0.778 | -0.961 | -0.622 |
|  |  |  |  |  |  |
| RT_20 vs Placebo_0 | | 0.276 |  |  |  |
| -> direct |  |  | -0.768 | -1.977 | 0.453 |
| -> indirect |  |  | -0.776 | -0.961 | -0.618 |
| -> MBNMA | |  | -0.77 | -0.941 | -0.617 |
|  |  |  |  |  |  |
| RT_16 vs Placebo_0 | | 0.347 |  |  |  |
| -> direct |  |  | -0.586 | -0.907 | -0.27 |
| -> indirect |  |  | -0.811 | -1.009 | -0.634 |
| -> MBNMA | |  | -0.759 | -0.919 | -0.607 |
|  |  |  |  |  |  |
| RT_10 vs Placebo_0 | | 0.243 |  |  |  |
| -> direct |  |  | -0.703 | -1.97 | 0.554 |
| -> indirect |  |  | -0.729 | -0.888 | -0.578 |
| -> MBNMA | |  | -0.725 | -0.878 | -0.574 |
|  |  |  |  |  |  |
| RT_4 vs Placebo_0 | | 0.315 |  |  |  |
| -> direct |  |  | -0.28 | -0.984 | 0.409 |
| -> indirect |  |  | -0.664 | -0.857 | -0.439 |
| -> MBNMA | |  | -0.63 | -0.815 | -0.413 |

**Table 11.2.3 Node-splitting analysis of inconsistency (Pain-number of repetitions/week)**

| **Comparison** | | **p-value** | **Median** | **2.50%** | **97.50%** |
| --- | --- | --- | --- | --- | --- |
| RT_750 vs RT_500 | | 0.051 |  |  |  |
| -> direct |  |  | -0.175 | -1.179 | 0.829 |
| -> indirect |  |  | -0.008 | -0.038 | 0 |
| -> MBNMA | |  | -0.008 | -0.04 | 0 |
|  |  |  |  |  |  |
| RT_500 vs RT_250 | | 0.085 |  |  |  |
| -> direct |  |  | -0.364 | -0.886 | 0.184 |
| -> indirect |  |  | -0.017 | -0.079 | -0.001 |
| -> MBNMA | |  | -0.023 | -0.1 | -0.001 |
|  |  |  |  |  |  |
| RT_150 vs RT_100 | | 0.092 |  |  |  |
| -> direct |  |  | -0.202 | -1.221 | 0.788 |
| -> indirect |  |  | -0.033 | -0.093 | -0.001 |
| -> MBNMA | |  | -0.032 | -0.094 | -0.002 |
|  |  |  |  |  |  |
| RT_1200 vs Placebo_0 | | 0.394 |  |  |  |
| -> direct |  |  | -0.653 | -1.396 | 0.058 |
| -> indirect |  |  | -0.799 | -1.004 | -0.626 |
| -> MBNMA | |  | -0.785 | -0.982 | -0.619 |
|  |  |  |  |  |  |
| RT_1000 vs Placebo_0 | | 0.325 |  |  |  |
| -> direct |  |  | -0.498 | -1.063 | 0.093 |
| -> indirect |  |  | -0.814 | -1.024 | -0.638 |
| -> MBNMA | |  | -0.782 | -0.974 | -0.619 |

**Table 11.2.4 Node-splitting analysis of inconsistency (Stiffness-intensity)**

| **Comparison** | | **p-value** | **Median** | **2.50%** | **97.50%** |
| --- | --- | --- | --- | --- | --- |
| RT_75 vs RT_70 | | 0.047 |  |  |  |
| -> direct |  |  | -0.224 | -0.853 | 0.392 |
| -> indirect |  |  | -0.014 | -0.03 | -0.001 |
| -> MBNMA | |  | -0.014 | -0.03 | -0.001 |
|  |  |  |  |  |  |
| RT_85 vs Placebo_0 | | 0.256 |  |  |  |
| -> direct |  |  | -0.146 | -0.783 | 0.472 |
| -> indirect |  |  | -0.571 | -0.804 | -0.362 |
| -> MBNMA | |  | -0.528 | -0.758 | -0.335 |
|  |  |  |  |  |  |
| RT_80 vs Placebo_0 | | 0.024 |  |  |  |
| -> direct |  |  | 0.274 | -0.274 | 0.868 |
| -> indirect |  |  | -0.554 | -0.754 | -0.384 |
| -> MBNMA | |  | -0.517 | -0.735 | -0.327 |
|  |  |  |  |  |  |
| RT_65 vs Placebo_0 | | 0.034 |  |  |  |
| -> direct |  |  | -1.248 | -1.818 | -0.619 |
| -> indirect |  |  | -0.402 | -0.568 | -0.245 |
| -> MBNMA | |  | -0.478 | -0.673 | -0.297 |
|  |  |  |  |  |  |
| RT_60 vs Placebo_0 | | 0.509 |  |  |  |
| -> direct |  |  | -0.327 | -0.716 | 0.049 |
| -> indirect |  |  | -0.498 | -0.713 | -0.299 |
| -> MBNMA | |  | -0.463 | -0.647 | -0.286 |
|  |  |  |  |  |  |
| RT_55 vs Placebo_0 | | 0.589 |  |  |  |
| -> direct |  |  | -0.422 | -0.9 | 0.086 |
| -> indirect |  |  | -0.443 | -0.635 | -0.26 |
| -> MBNMA | |  | -0.444 | -0.623 | -0.274 |
|  |  |  |  |  |  |
| RT_50 vs Placebo_0 | | 0.085 |  |  |  |
| -> direct |  |  | -0.933 | -1.452 | -0.45 |
| -> indirect |  |  | -0.376 | -0.548 | -0.22 |
| -> MBNMA | |  | -0.425 | -0.603 | -0.258 |
|  |  |  |  |  |  |
| RT_40 vs Placebo_0 | | 0.297 |  |  |  |
| -> direct |  |  | -0.651 | -1.57 | 0.311 |
| -> indirect |  |  | -0.366 | -0.562 | -0.214 |
| -> MBNMA | |  | -0.378 | -0.572 | -0.223 |
|  |  |  |  |  |  |
| RT_30 vs Placebo_0 | | 0.148 |  |  |  |
| -> direct |  |  | -0.989 | -1.966 | -0.023 |
| -> indirect |  |  | -0.292 | -0.504 | -0.17 |
| -> MBNMA | |  | -0.318 | -0.532 | -0.18 |

**Table 11.2.5 Node-splitting analysis of inconsistency (Stiffness-period)**

| **Comparison** | | **p-value** | **Median** | **2.50%** | **97.50%** |
| --- | --- | --- | --- | --- | --- |
| RT_24 vs Placebo_0 | | 0.162 |  |  |  |
| -> direct |  |  | -0.143 | -0.82 | 0.531 |
| -> indirect |  |  | -0.923 | -1.377 | -0.504 |
| -> MBNMA | |  | -0.706 | -1.102 | -0.399 |
|  |  |  |  |  |  |
| RT_20 vs Placebo_0 | | 0.374 |  |  |  |
| -> direct |  |  | -0.991 | -2.039 | 0.077 |
| -> indirect |  |  | -0.59 | -0.936 | -0.313 |
| -> MBNMA | |  | -0.634 | -0.951 | -0.368 |
|  |  |  |  |  |  |
| RT_16 vs Placebo_0 | | 0.089 |  |  |  |
| -> direct |  |  | -0.16 | -0.519 | 0.218 |
| -> indirect |  |  | -0.688 | -0.958 | -0.431 |
| -> MBNMA | |  | -0.553 | -0.804 | -0.322 |
|  |  |  |  |  |  |
| RT_10 vs Placebo_0 | | 0.118 |  |  |  |
| -> direct |  |  | -1.287 | -2.483 | -0.125 |
| -> indirect |  |  | -0.382 | -0.585 | -0.205 |
| -> MBNMA | |  | -0.402 | -0.601 | -0.222 |
|  |  |  |  |  |  |
| RT_4 vs Placebo_0 | | 0.331 |  |  |  |
| -> direct |  |  | -0.339 | -1.281 | 0.642 |
| -> indirect |  |  | -0.184 | -0.479 | -0.089 |
| -> MBNMA | |  | -0.186 | -0.464 | -0.095 |

**Table 11.2.6 Node-splitting analysis of inconsistency (Stiffness-number of repetitions/week)**

| **Comparison** | **p-value** | **Median** | **2.50%** | **97.50%** |
| --- | --- | --- | --- | --- |
| RT_750 vs RT_500 | 0.111 |  |  |  |
| -> direct |  | 0.021 | -0.733 | 0.835 |
| -> indirect |  | -0.034 | -0.08 | -0.004 |
| -> MBNMA | | -0.034 | -0.079 | -0.005 |
|  |  |  |  |  |
| RT_500 vs RT_250 | 0.051 |  |  |  |
| -> direct |  | -0.568 | -1.047 | -0.084 |
| -> indirect |  | -0.064 | -0.146 | -0.006 |
| -> MBNMA | | -0.082 | -0.163 | -0.013 |
|  |  |  |  |  |
| RT_1200 vs Placebo_0 | 0.015 |  |  |  |
| -> direct |  | -1.964 | -2.862 | -1.066 |
| -> indirect |  | -0.549 | -0.777 | -0.35 |
| -> MBNMA | | -0.639 | -0.892 | -0.416 |
|  |  |  |  |  |
| RT_150 vs Placebo_0 | 0.373 |  |  |  |
| -> direct |  | -0.613 | -1.172 | -0.047 |
| -> indirect |  | -0.394 | -0.58 | -0.256 |
| -> MBNMA | | -0.412 | -0.579 | -0.269 |
|  |  |  |  |  |
| RT_100 vs Placebo_0 | 0.672 |  |  |  |
| -> direct |  | -0.282 | -0.564 | -0.001 |
| -> indirect |  | -0.367 | -0.621 | -0.209 |
| -> MBNMA | | -0.344 | -0.524 | -0.215 |

**Table 11.2.7 Node-splitting analysis of inconsistency (Function-intensity)**

| **Comparison** | | **p-value** | **Median** | **2.50%** | **97.50%** |
| --- | --- | --- | --- | --- | --- |
| RT_75 vs RT_70 | | 0.05 |  |  |  |
| -> direct |  |  | 0.073 | -0.864 | 1.042 |
| -> indirect |  |  | 0.015 | 0 | 0.042 |
| -> MBNMA | |  | 0.015 | 0 | 0.042 |
|  |  |  |  |  |  |
| RT_85 vs Placebo_0 | | 0.101 |  |  |  |
| -> direct |  |  | 0.076 | -0.659 | 0.82 |
| -> indirect |  |  | 0.889 | 0.629 | 1.187 |
| -> MBNMA | |  | 0.797 | 0.561 | 1.071 |
|  |  |  |  |  |  |
| RT_80 vs Placebo_0 | | 0.087 |  |  |  |
| -> direct |  |  | -0.547 | -2.134 | 1.017 |
| -> indirect |  |  | 0.812 | 0.583 | 1.071 |
| -> MBNMA | |  | 0.783 | 0.551 | 1.039 |
|  |  |  |  |  |  |
| RT_65 vs Placebo_0 | | 0.459 |  |  |  |
| -> direct |  |  | 0.748 | -0.085 | 1.604 |
| -> indirect |  |  | 0.739 | 0.491 | 0.979 |
| -> MBNMA | |  | 0.735 | 0.513 | 0.963 |
|  |  |  |  |  |  |
| RT_60 vs Placebo_0 | | 0.165 |  |  |  |
| -> direct |  |  | 1.127 | 0.639 | 1.61 |
| -> indirect |  |  | 0.623 | 0.382 | 0.858 |
| -> MBNMA | |  | 0.716 | 0.495 | 0.937 |
|  |  |  |  |  |  |
| RT_55 vs Placebo_0 | | 0.377 |  |  |  |
| -> direct |  |  | 0.411 | -0.228 | 1.06 |
| -> indirect |  |  | 0.73 | 0.496 | 0.962 |
| -> MBNMA | |  | 0.694 | 0.475 | 0.914 |
|  |  |  |  |  |  |
| RT_50 vs Placebo_0 | | 0.192 |  |  |  |
| -> direct |  |  | 1.21 | 0.551 | 1.917 |
| -> indirect |  |  | 0.648 | 0.412 | 0.907 |
| -> MBNMA | |  | 0.669 | 0.447 | 0.892 |
|  |  |  |  |  |  |
| RT_40 vs Placebo_0 | | 0.022 |  |  |  |
| -> direct |  |  | 2.306 | 1.029 | 3.634 |
| -> indirect |  |  | 0.568 | 0.366 | 0.816 |
| -> MBNMA | |  | 0.611 | 0.39 | 0.865 |
|  |  |  |  |  |  |
| RT_35 vs Placebo_0 | | 0.338 |  |  |  |
| -> direct |  |  | 0.294 | -1 | 1.576 |
| -> indirect |  |  | 0.589 | 0.356 | 0.882 |
| -> MBNMA | |  | 0.577 | 0.357 | 0.854 |
|  |  |  |  |  |  |
| RT_30 vs Placebo_0 | | 0.129 |  |  |  |
| -> direct |  |  | 1.363 | 0.431 | 2.344 |
| -> indirect |  |  | 0.477 | 0.288 | 0.767 |
| -> MBNMA | |  | 0.536 | 0.319 | 0.839 |
|  |  |  |  |  |  |
| RT_10 vs Placebo_0 | | 0.315 |  |  |  |
| -> direct |  |  | 0.822 | -0.446 | 2.144 |
| -> indirect |  |  | 0.24 | 0.116 | 0.703 |
| -> MBNMA | |  | 0.269 | 0.122 | 0.749 |

**Table 11.2.8 Node-splitting analysis of inconsistency (Function-period)**

| **Comparison** | | **p-value** | **Median** | **2.50%** | **97.50%** |
| --- | --- | --- | --- | --- | --- |
| RT_72 vs Placebo_0 | | 0.136 |  |  |  |
| -> direct |  |  | 0.097 | -0.74 | 0.964 |
| -> indirect |  |  | 1.087 | 0.68 | 3.064 |
| -> MBNMA | |  | 0.846 | 0.584 | 1.245 |
|  |  |  |  |  |  |
| RT_24 vs Placebo_0 | | 0.169 |  |  |  |
| -> direct |  |  | 0.211 | -0.56 | 0.98 |
| -> indirect |  |  | 0.868 | 0.614 | 1.166 |
| -> MBNMA | |  | 0.798 | 0.562 | 1.054 |
|  |  |  |  |  |  |
| RT_20 vs Placebo_0 | | 0.312 |  |  |  |
| -> direct |  |  | 0.597 | -0.826 | 2.081 |
| -> indirect |  |  | 0.787 | 0.544 | 1.056 |
| -> MBNMA | |  | 0.785 | 0.553 | 1.03 |
|  |  |  |  |  |  |
| RT_16 vs Placebo_0 | | 0.168 |  |  |  |
| -> direct |  |  | 0.34 | -0.175 | 0.829 |
| -> indirect |  |  | 0.849 | 0.61 | 1.111 |
| -> MBNMA | |  | 0.764 | 0.539 | 0.995 |
|  |  |  |  |  |  |
| RT_10 vs Placebo_0 | | 0.261 |  |  |  |
| -> direct |  |  | 0.656 | -0.954 | 2.254 |
| -> indirect |  |  | 0.71 | 0.472 | 0.934 |
| -> MBNMA | |  | 0.712 | 0.474 | 0.933 |
|  |  |  |  |  |  |
| RT_4 vs Placebo_0 | | 0.27 |  |  |  |
| -> direct |  |  | -0.036 | -1.264 | 1.352 |
| -> indirect |  |  | 0.597 | 0.293 | 0.875 |
| -> MBNMA | |  | 0.576 | 0.292 | 0.853 |

**Table 11.2.9 Node-splitting analysis of inconsistency (Function-number of repetition/week)**

| **Comparison** | | **p-value** | **Median** | **2.50%** | **97.50%** |
| --- | --- | --- | --- | --- | --- |
| RT_750 vs RT_500 | | 0.048 |  |  |  |
| -> direct |  |  | 0.277 | -1.038 | 1.54 |
| -> indirect |  |  | 0.008 | 0 | 0.047 |
| -> MBNMA | |  | 0.008 | 0 | 0.045 |
|  |  |  |  |  |  |
| RT_500 vs RT_250 | | 0.101 |  |  |  |
| -> direct |  |  | 0.348 | -0.469 | 1.158 |
| -> indirect |  |  | 0.018 | 0.001 | 0.096 |
| -> MBNMA | |  | 0.023 | 0.001 | 0.109 |
|  |  |  |  |  |  |
| RT_1200 vs Placebo_0 | | 0.275 |  |  |  |
| -> direct |  |  | 0.296 | -0.989 | 1.537 |
| -> indirect |  |  | 0.791 | 0.553 | 1.045 |
| -> MBNMA | |  | 0.768 | 0.542 | 1.022 |
|  |  |  |  |  |  |
| RT_1000 vs Placebo_0 | | 0.477 |  |  |  |
| -> direct |  |  | 0.619 | -0.084 | 1.364 |
| -> indirect |  |  | 0.787 | 0.549 | 1.047 |
| -> MBNMA | |  | 0.766 | 0.541 | 1.015 |
|  |  |  |  |  |  |
| RT_150 vs Placebo_0 | | 0.45 |  |  |  |
| -> direct |  |  | 0.903 | 0.312 | 1.505 |
| -> indirect |  |  | 0.655 | 0.406 | 0.905 |
| -> MBNMA | |  | 0.685 | 0.456 | 0.907 |
|  |  |  |  |  |  |
| RT_100 vs Placebo_0 | | 0.119 |  |  |  |
| -> direct |  |  | 1.105 | 0.628 | 1.585 |
| -> indirect |  |  | 0.513 | 0.268 | 0.78 |
| -> MBNMA | |  | 0.652 | 0.394 | 0.885 |

Supplementary 12: Non-linear functions and models fit comparison

The different doses of resistance training were meta-analysed as independent and unrelated treatments (i.e., “split” NMA). This step is useful to determine which function fits the data better and should subsequently be used in a Model-Based Network Meta-Analysis (MBNMA) [6]. Figure 11.1-9 show the different responses of each dose for resistance training, respectively.

**Figure 12.1.** “Split” NMA of different resistance training intensity (Pain). RT resistance training.

**Figure 12.2.** “Split” NMA of different resistance training period (Pain). RT resistance training.

**Figure 12.3.** “Split” NMA of different resistance training number of repetitions/week (Pain). RT resistance training.

**Figure 12.4.** “Split” NMA of different resistance training intensity (Stiffness). RT resistance training.

**Figure 12.5.** “Split” NMA of different resistance training period (Stiffness). RT resistance training.

**Figure 12.6.** “Split” NMA of different resistance training number of repetition/week (Stiffness). RT resistance training.

**Figure 12.7.** “Split” NMA of different resistance training intensity (Function). RT resistance training.

**Figure 12.8.** “Split” NMA of different resistance training period (Function). RT resistance training.

**Figure 12.9.** “Split” NMA of different resistance training number of repetition/week (Function). RT resistance training

Table 11 shows the fit indices from each of the models fitted. Our results show that the best-fitting model for different outcome measures at different specific doses is restricted cubic splines. DIC = Deviance Information Criterion; SD = Between-study Standard Deviation; pD: Number of estimated parameters; NA = Not Applicable. The SD is presented as the main value.

**Table 12.1.** Models fit comparison (Pian-intensity)

| Model | DIC | SD | Deviance | Residual deviance | pD |
| --- | --- | --- | --- | --- | --- |
| Emax  (common treatment effects) | 479.0 | NA | 430.268 | 266.805 | 49.4 |
| Emax  (RANDOM treatment effects) | 359.6 | 0.445 | 271.477 | 108.013 | 88.9 |
| Linear  (common treatment effects) | 506.4 | NA | 458.473 | 295.010 | 48.7 |
| Linear  (random treatment effects) | 362.6 | 0.486 | 272.484 | 109.021 | 91.1 |
| EXponential  (common treatment effects) | 477.8 | NA | 429.512 | 266.049 | 48.8 |
| EXponential  (RANDOM treatment effects) | 360.5 | 0.431 | 271.972 | 108.509 | 89.0 |
| Restricted cubic spline  (common treatment effects; 3 knots) | 460.2 | NA | 410.960 | 247.497 | 49.8 |
| Restricted cubic spline  (random treatment effects; 3 knots) | 358.0 | 0.412 | 271.314 | 107.851 | 87.6 |
| Non-parametric monotonically up  (common treatment effects) | 777.2 | NA | 729.808 | 566.345 | 48.1 |
| Non-parametric monotonically up  (RANDOM treatment effects) | 375.1 | 3.068 | 281.593 | 118.130 | 94.1 |

**Table 12.2.** Models fit comparison (Pian-period)

| Model | DIC | SD | Deviance | Residual deviance | pD |
| --- | --- | --- | --- | --- | --- |
| Emax  (common treatment effects) | 479.6 | NA | 431.145 | 267.682 | 49.2 |
| Emax  (RANDOM treatment effects) | 360.3 | 0.445 | 271.498 | 108.035 | 89.4 |
| Linear  (common treatment effects) | 715.2 | NA | 666.952 | 503.489 | 49.0 |
| Linear  (random treatment effects) | 367.7 | 0.743 | 270.814 | 107.351 | 97.7 |
| EXponential  (common treatment effects) | 477.3 | NA | 429.246 | 265.782 | 48.9 |
| EXponential  (RANDOM treatment effects) | 359.5 | 0.431 | 271.441 | 107.977 | 88.7 |
| Restricted cubic spline  (common treatment effects; 3 knots) | 518.6 | NA | 469.371 | 305.908 | 49.8 |
| Restricted cubic spline  (random treatment effects; 3 knots) | 354.4 | 0.408 | 271.783 | 107.320 | 93.3 |
| Non-parametric monotonically up  (common treatment effects) | 776.0 | NA | 728.194 | 564.731 | 48.5 |
| Non-parametric monotonically up  (RANDOM treatment effects) | 373.8 | 3.070 | 280.613 | 117.150 | 94.1 |

**Table 12.3.** Models fit comparison (Pian-number of repetitions/week).

| Model | DIC | SD | Deviance | Residual deviance | pD |
| --- | --- | --- | --- | --- | --- |
| Emax  (common treatment effects) | 479.2 | NA | 430.973 | 267.509 | 48.9 |
| Emax  (RANDOM treatment effects) | 360.4 | 0.445 | 271.524 | 108.061 | 89.7 |
| Linear  (common treatment effects) | 644.6 | NA | 596.535 | 433.072 | 48.8 |
| Linear  (random treatment effects) | 366.3 | 0.669 | 270.434 | 106.971 | 96.4 |
| EXponential  (common treatment effects) | 477.8 | NA | 429.443 | 265.980 | 48.9 |
| EXponential  (RANDOM treatment effects) | 359.0 | 0.431 | 271.426 | 107.962 | 88.1 |
| Restricted cubic spline  (common treatment effects; 3 knots) | 574.2 | NA | 524.923 | 361.460 | 49.9 |
| Restricted cubic spline  (random treatment effects; 3 knots) | 355.2 | 0.387 | 270.152 | 107.689 | 94.9 |
| Non-parametric monotonically up  (common treatment effects) | 768.5 | NA | 720.997 | 557.534 | 48.0 |
| Non-parametric monotonically up  (RANDOM treatment effects) | 374.1 | 2.725 | 281.973 | 118.510 | 92.9 |

**Table 12.4.** Models fit comparison (Stiffness-intensity)

| Model | DIC | SD | Deviance | Residual deviance | pD |
| --- | --- | --- | --- | --- | --- |
| Emax  (common treatment effects) | 193.5 | NA | 166.084 | 92.425 | 28.1 |
| Emax  (RANDOM treatment effects) | 182.5 | 0.318 | 137.984 | 64.325 | 45.0 |
| Linear  (common treatment effects) | 199.8 | NA | 172.587 | 98.928 | 27.8 |
| Linear  (random treatment effects) | 182.7 | 0.359 | 137.076 | 63.417 | 46.2 |
| EXponential  (common treatment effects) | 190.1 | NA | 162.936 | 89.277 | 27.9 |
| EXponential  (RANDOM treatment effects) | 180.2 | 0.295 | 137.839 | 64.180 | 43.2 |
| Restricted cubic spline  (common treatment effects; 3 knots) | 184.8 | NA | 156.262 | 82.604 | 29.1 |
| Restricted cubic spline  (random treatment effects; 3 knots) | 179.1 | 0.253 | 137.588 | 63.929 | 42.1 |
| Non-parametric monotonically up  (common treatment effects) | 262.1 | NA | 235.204 | 161.545 | 27.5 |
| Non-parametric monotonically up  (RANDOM treatment effects) | 204.2 | 2.043 | 154.541 | 80.882 | 50.5 |

**Table 12.5.** Models fit comparison (Stiffness-period)

| Model | DIC | SD | Deviance | Residual deviance | pD |
| --- | --- | --- | --- | --- | --- |
| Emax  (common treatment effects) | 194.6 | NA | 167.424 | 93.766 | 28.1 |
| Emax  (RANDOM treatment effects) | 181.6 | 0.370 | 136.605 | 62.946 | 45.9 |
| Linear  (common treatment effects) | 208.1 | NA | 181.161 | 107.502 | 27.7 |
| Linear  (random treatment effects) | 184.1 | 0.405 | 136.962 | 63.303 | 47.7 |
| EXponential  (common treatment effects) | 190.1 | NA | 163.001 | 89.342 | 27.9 |
| EXponential  (RANDOM treatment effects) | 180.7 | 0.295 | 137.672 | 64.013 | 43.7 |
| Restricted cubic spline  (common treatment effects; 3 knots) | 189.7 | NA | 161.354 | 87.695 | 29.1 |
| Restricted cubic spline  (random treatment effects; 3 knots) | 180.7 | 0.294 | 137.570 | 63.911 | 43.9 |
| Non-parametric monotonically up  (common treatment effects) | 262.1 | NA | 235.248 | 161.589 | 27.4 |
| Non-parametric monotonically up  (RANDOM treatment effects) | 203.7 | 1.961 | 154.289 | 80.630 | 50.0 |

**Table 12.6.** Models fit comparison (Stiffness-number of repetition/week)

| Model | DIC | SD | Deviance | Residual deviance | pD |
| --- | --- | --- | --- | --- | --- |
| Emax  (common treatment effects) | 186.2 | NA | 158.826 | 85.167 | 28.1 |
| Emax  (RANDOM treatment effects) | 179.0 | 0.270 | 137.815 | 64.157 | 41.7 |
| Linear  (common treatment effects) | 189.5 | NA | 162.180 | 88.521 | 28.1 |
| Linear  (random treatment effects) | 178.3 | 0.290 | 136.334 | 62.675 | 42.5 |
| EXponential  (common treatment effects) | 190.2 | NA | 162.911 | 89.252 | 28.0 |
| EXponential  (RANDOM treatment effects) | 180.8 | 0.294 | 138.073 | 64.414 | 43.5 |
| Restricted cubic spline  (common treatment effects; 3 knots) | 191.8 | NA | 163.340 | 89.681 | 29.1 |
| Restricted cubic spline  (random treatment effects; 3 knots) | 179.2 | 0.302 | 136.047 | 62.388 | 43.7 |
| Non-parametric monotonically up  (common treatment effects) | 258.9 | NA | 232.494 | 158.835 | 26.9 |
| Non-parametric monotonically up  (RANDOM treatment effects) | 203.8 | 1.693 | 155.205 | 81.546 | 49.4 |

**Table 12.7.** Models fit comparison (Function-intensity)

| Model | DIC | SD | Deviance | Residual deviance | pD |
| --- | --- | --- | --- | --- | --- |
| Emax  (common treatment effects) | 615.1 | NA | 575.917 | 269.840 | 39.7 |
| Emax  (RANDOM treatment effects) | 464.1 | 0.612 | 390.082 | 84.005 | 74.7 |
| Linear  (common treatment effects) | 663.1 | NA | 623.871 | 317.794 | 40.0 |
| Linear  (random treatment effects) | 465.0 | 0.669 | 390.170 | 84.094 | 75.4 |
| EXponential  (common treatment effects) | 612.6 | NA | 573.590 | 267.513 | 39.7 |
| EXponential  (RANDOM treatment effects) | 464.0 | 0.577 | 390.634 | 84.558 | 73.7 |
| Restricted cubic spline  (common treatment effects; 3 knots) | 581.7 | NA | 541.039 | 234.963 | 41.4 |
| Restricted cubic spline  (random treatment effects; 3 knots) | 461.6 | 0.530 | 389.532 | 83.455 | 72.9 |
| Non-parametric monotonically up  (common treatment effects) | 590.1 | NA | 551.445 | 245.368 | 39.4 |
| Non-parametric monotonically up  (RANDOM treatment effects) | 464.8 | 5.918 | 394.128 | 88.052 | 71.4 |

**Table 12.8.** Models fit comparison (Function-period)

| Model | DIC | SD | Deviance | Residual deviance | pD |
| --- | --- | --- | --- | --- | --- |
| Emax  (common treatment effects) | 615.4 | NA | 575.463 | 269.386 | 40.2 |
| Emax  (RANDOM treatment effects) | 464.0 | 0.606 | 390.123 | 84.046 | 74.4 |
| Linear  (common treatment effects) | 789.0 | NA | 749.803 | 443.727 | 40.0 |
| Linear  (random treatment effects) | 466.7 | 0.857 | 389.723 | 83.646 | 78.0 |
| EXponential  (common treatment effects) | 613.1 | NA | 573.520 | 267.444 | 40.0 |
| EXponential  (RANDOM treatment effects) | 463.1 | 0.579 | 390.311 | 84.234 | 73.7 |
| Restricted cubic spline  (common treatment effects; 3 knots) | 656.5 | NA | 615.837 | 309.760 | 41.2 |
| Restricted cubic spline  (random treatment effects; 3 knots) | 455.6 | 0.487 | 378.321 | 83.245 | 71.9 |
| Non-parametric monotonically up  (common treatment effects) | 583.7 | NA | 545.382 | 239.305 | 39.0 |
| Non-parametric monotonically up  (RANDOM treatment effects) | 463.6 | 5.911 | 393.677 | 87.600 | 70.7 |

**Table 12.9.** Models fit comparison (Function-number of repetition/week)

| Model | DIC | SD | Deviance | Residual deviance | pD |
| --- | --- | --- | --- | --- | --- |
| Emax  (common treatment effects) | 615.0 | NA | 575.591 | 269.515 | 40.2 |
| Emax  (RANDOM treatment effects) | 463.7 | 0.601 | 390.194 | 84.117 | 74.1 |
| Linear  (common treatment effects) | 740.7 | NA | 701.684 | 395.607 | 39.8 |
| Linear  (random treatment effects) | 466.6 | 0.810 | 389.529 | 83.452 | 77.6 |
| EXponential  (common treatment effects) | 612.8 | NA | 573.479 | 267.403 | 39.8 |
| EXponential  (RANDOM treatment effects) | 463.2 | 0.578 | 390.085 | 84.008 | 74.0 |
| Restricted cubic spline  (common treatment effects; 3 knots) | 712.7 | NA | 672.302 | 366.225 | 41.1 |
| Restricted cubic spline  (random treatment effects; 3 knots) | 455.8 | 0.484 | 379.825 | 83.748 | 76.8 |
| Non-parametric monotonically up  (common treatment effects) | 583.1 | NA | 543.528 | 237.451 | 40.2 |
| Non-parametric monotonically up  (RANDOM treatment effects) | 464.0 | 5.679 | 393.417 | 87.341 | 87.341 |

Further to model fit indices, deviance plots showing the contribution of each data point to the residual deviance are also useful to confirm the robustness of model selection [6]. Each data point should contribute about 1 to the posterior mean deviance, which indicates good model fit [7]. The deviance plot for treatment effects (Supplementary 11, Figure 11) confirm the robustness of our model selection.

Figure 12.1 Deviance plots at treatment-level (Pain-intensity). RT resistance training.

Figure 12.2 Deviance plots at treatment-level (Pain-period). RT resistance training.

Figure 12.3 Deviance plots at treatment-level (Pain-number of repetitions/week). RT resistance training.

Figure 12.4 Deviance plots at treatment-level (stiffness-intensity). RT resistance training.

Figure 12.5 Deviance plots at treatment-level (stiffness-period). RT resistance training.

Figure 12.6 Deviance plots at treatment-level (stiffness-number of repetition/week). RT resistance training.

Figure 12.7 Deviance plots at treatment-level (function-intensity). RT resistance training.

Figure 12.8 Deviance plots at treatment-level (function-period). RT resistance training.

Figure 12.9 Deviance plots at treatment-level (function-number of repetition/week). RT resistance training

**References**

1. Goh S-L, Persson MSM, Stocks J, et al. Relative Efficacy of Different Exercises for Pain, Function, Performance and Quality of Life in Knee and Hip Osteoarthritis: Systematic Review and Network Meta-Analysis. *Sports Med.* 2019;49(5):743-761. doi: 10.1007/s40279-019-01082-0.

2. Kellgren JH, Lawrence JS. Radiological assessment of osteo-arthrosis. *Ann Rheum Dis.* 1957;16(4):494-502. doi. <https://pubmed.ncbi.nlm.nih.gov/13498604>.

3. Ter Veer E, van Oijen MGH, van Laarhoven HWM. The Use of (Network) Meta-Analysis in Clinical Oncology. *Front Oncol.* 2019;9:822. doi: 10.3389/fonc.2019.00822.

4. Wheeler DC, Hickson DA, Waller LA. Assessing Local Model Adequacy in Bayesian Hierarchical Models Using the Partitioned Deviance Information Criterion. *Comput Stat Data Anal.* 2010;54(6):1657-1671. doi. <https://pubmed.ncbi.nlm.nih.gov/21243121>.

5. van Valkenhoef G, Dias S, Ades AE, Welton NJ. Automated generation of node-splitting models for assessment of inconsistency in network meta-analysis. *Res Synth Methods.* 2016;7(1):80-93. doi: 10.1002/jrsm.1167.

6. Pedder H. MBNMAdose: An R package for incorporating dose-response information into Network Meta-Analysis. Paper presented at: Evidence Synthesis and Meta-Analysis in R Conference 20212021.

7. Dias S, Sutton AJ, Ades AE, Welton NJ. Evidence synthesis for decision making 2: a generalized linear modeling framework for pairwise and network meta-analysis of randomized controlled trials. *Medical Decision Making : an International Journal of the Society For Medical Decision Making.* 2013;33(5):607-617. doi: 10.1177/0272989X12458724.
